# Synthesis, characterization, and antibacterial activity studies of two Co(II) complexes with 2-[(*E*)-(3-acetyl-4-hydroxyphenyl)diazenyl]-4-(2-hydroxyphenyl)thiophene-3-carboxylic acid as a ligand

**DOI:** 10.1186/s13065-024-01179-2

**Published:** 2024-04-16

**Authors:** Emmanuel Sopbué Fondjo, Sorelle Songmi Feuze, Jean-de-Dieu Tamokou, Apollinaire Tsopmo, Giscard Doungmo, Peter Simon Friedrich Wilhelm, Donald Léonel Feugap Tsamo, Bruno Lenta Ndjakou, Jules Roger Kuiate

**Affiliations:** 1 Laboratory of Applied Synthetic Organic Chemistry, Department of Chemistry, Faculty of Science, University of Dschang, P.O. Box 67, Dschang, Republic of Cameroon; 2 Research Unit of Microbiology and Antimicrobial Substances, Department of Biochemistry, Faculty of Science, University of Dschang, PO Box 067, Dschang, Republic of Cameroon; 3 Department of Chemistry, Carleton University, 1125 Colonel By Drive, Ottawa, K1S 5B6 Canada; 4 Institut für Anorganische Chemie, Christian-Albrechts-Universität zu Kiel, Max-Eyth-Str. 2, 24118 Kiel, Germany; 5 Polymer Chemistry Laboratory, Faculty of Live Sciences, Rhine-Waal University of Applied Sciences, Campus Kleve, Marie-Curie Strasse 1, 47533 Kleve, Germany; 6 Higher Teacher’s Training College, University of Yaounde I, P. O. Box 47, Yaounde, Cameroon

**Keywords:** Azoic ligand, Cobalt(II) complexes, DFT, Anti-bacterial activity

## Abstract

Two new Cobalt(II) complexes **12** and **13** have been synthesized from 2-[(*E*)-(3-acetyl-4-hydroxyphenyl)diazenyl]-4-(2-hydroxyphenyl)thiophene-3-carboxylic acid (**11**) as a novel ligand. These three new compounds were characterized on the basis of their powder X-Ray Diffraction, UV–Vis, IR, NMR, elemental analysis and MS spectral data. DFT/B3LYP mode of calculations were carried out to determine some theorical parameters of the molecular structure of the ligand. The purity of the azoic ligand and the metal complexes were ascertained by TLC and melting points. The analysis of the IR spectra of the polyfunctionalized azo compound **11** and its metal complexes **12** and **13**, reveals that the coordination patterns of the ligand are hexadentate and tetradentate respectively. Based on the UV–Vis electronic spectral data and relevant literature reports**,** the ligand and derived complexes were assigned the *E* (*trans*) isomer form. Likewise, octahedral and square-planar geometries were respectively assigned to the cobalt(II) complexes. The broth microdilution method was used for antibacterial assays through the determination of minimum inhibitory concentration (MIC) and minimum bactericidal concentration (MBC). The ligand **11** displayed moderate antibacterial activity (MIC = 32–128 μg/mL) against *Staphylococcus aureus* ATCC25923*, Escherichia coli* ATCC25922, *Pseudomonas aeruginosa* and *Klebsiella pneumoniae* 22. The octahedral cobalt(II) complex **12** showed moderate activity against *Pseudomonas aeruginosa* (MIC = 128 μg/mL) and *Klebsiella pneumoniae* 22 (MIC = 64 μg/mL) and none against *Staphylococcus aureus* ATCC25923 and *Escherichia coli* ATCC25922, whereas the square-planar complex **13** displayed moderate activity only on *Klebsiella pneumoniae* 22 (MIC = 64 μg/mL)**.**

**Graphical abstract:**

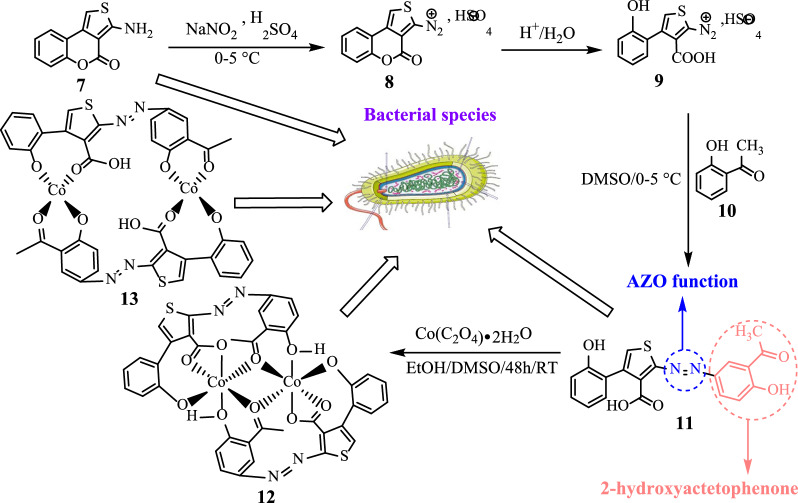

**Supplementary Information:**

The online version contains supplementary material available at 10.1186/s13065-024-01179-2.

## Introduction

**Scheme 1:**
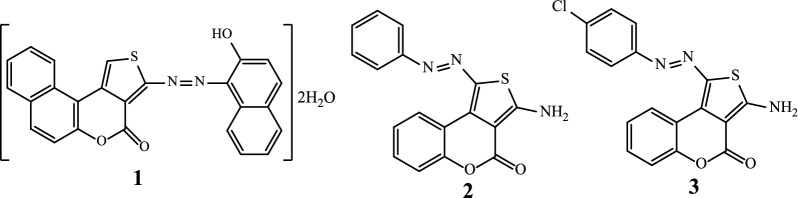
Structures of some azo thiophenes compounds

Antimicrobial resistance (AMR) appears over time as a phenomenon mainly linked to the genetic evolution of pathogens. The direct consequence of this AMR is that it makes infections more difficult to treat [1–3]. There is therefore a constant search for new antimicrobial compounds from natural sources [4–7] or via synthetic routes [8–10] as possible solutions. Thus, among the broad range of bioactive molecules of synthetic origin, coordination compounds in general and those based on hybrid heterocyclic ligands such as azo thiophenes scaffolds such as **1**, **2** and **3** (Scheme 1) in particular [11, 12], represent the most promising molecules for the discovery of novel antimicrobial drugs [13, 14].

Such hybrid molecules are expected to combine the properties of the chelating heterocyclic ligands with those of the central metal ions and to exhibit much better biological profiles [15–18].

**Scheme 2:**
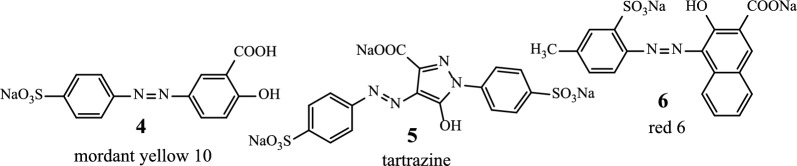
Structures of some industrial azo dyes

Azo compounds have a long history and are important part of our daily life. They are mainly used as dyes and pigments in various fields, such as: textile dyeing (mordant yellow 10 (**4**) [19]); the food (tartrazine (**5**) [20]) and cosmetics (red 6 (**6**) [21]) industries (Scheme 2).

They also have many other applications in physicochemistry, analysis, catalysis [22, 23] and pharmacy [24] because of their special complexing abilities, sensitivity as chromogenic reagents, usage in spectrophotometry and ability to detect a variety of metal ions. In addition, they have been of major importance in drug development due to their antioxidant, anti-inflammatory, fungicidal, antidiabetic, bacteriostatic, and antiseptic activities [25–27]. These compounds and their derivatives have some potential applications in different fields, including industrial and biological research [28, 29]. For instance, in the dyeing of wool and synthetic polyamides, the azo complexes of Cr(III) and Co(III) are extensively utilized in industry [30], as well as the azo complexes of Ni(II) and Cu(II), which are utilized in biology as antibacterial and anticancer medications [31, 32]. Azo compounds having in their structures both the thiophenic and phenolic fragments (which separately have each amazing antiviral, antibacterial, antifungal, cytotoxic [33], antioxidant and antiradical [34] properties), are expected to combine the different properties of the latter in the hybrid structures [35].

Cobalt is the chemical element with atomic number 27, symbol Co and electronic structure [Ar]4s^2^3d^7^ belonging to block “d” of the periodic table of elements. It is relatively rare, gray in color, ductile, fragile and magnetic. Relatively unreactive, it does not oxidize in humid or dry air at normal environmental temperatures. The two valence states, cobaltous(II) and cobaltic(III), melt at 1493 °C with limited water solubility. These properties are similar to those of iron and nickel, which are neighbors in the periodic table [36]. It is one of the most important transition metals from a biological point of view. Its ions act in the activation of cholinesterase and provide protection against excessive oxygen pressure in the lungs during respiration. They also act as bacteriostatic agents comparable with antibiotics [37]. The cobalt ion is an integral part of the vitamin B12 molecule [38] which has a key role in the maturation of red blood cells, the chemical name of this vitamin, cobalamin, also evokes the importance of the cobalt which is present in it at 4% [39, 40]. Cobalt complexes have also been suggested to possess antirheumatic, antihistamine [41, 42] antifungals and antivirals properties [43]. Cobalt coordination compounds are the earliest known metal complexes and coordination chemistry was founded with the study of these promising compounds [44].

As part of a continuing interest in the chemistry and biological properties of azo compounds having in their structures thiophenic and phenolic fragments, we have undertaken in this study to determine how they coordinate with cobalt(II) in order to evaluate the antimicrobial activities of the synthesized product and those of their cobalt(II) complexes as well, on certain resistant bacterial strains.

## Results and discussion

### Chemistry

**Scheme 3:**
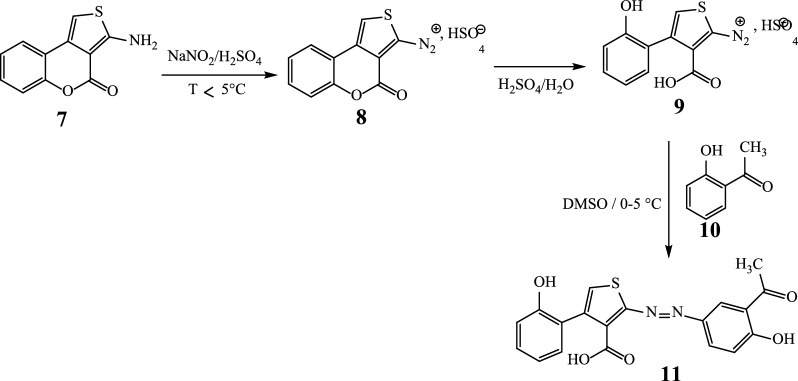
Reaction sequences for the preparation of compound **11**

The new azoic ligand **11** was prepared using the thienocoumarin **7** as starting material. Procedure for the preparation of **7** has been reported earlier [45, 46]. The general preparation process of **11** is displayed in Scheme 3 [46, 47].

**Scheme 4:**
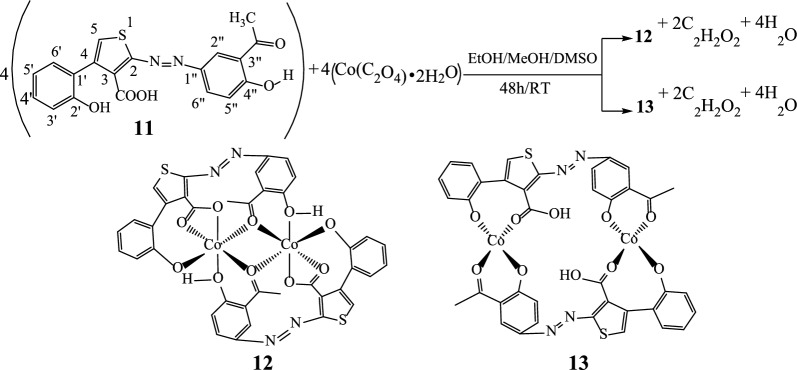
Reaction sequences to the complexes **12** and **13** ([Co_2_(C_19_H_12_N_2_O_5_S)_2_])

The structure of substrate ligand **11** (C_19_H_14_N_2_O_5_S) was confirmed with its physical and spectroscopic data. Reaction of compound **11** (previously dissolved in 4 mL of DMSO) with Co(C_2_O_4_)‧2H_2_O (dissolves in EtOH/MeOH 2:1) with constant stirring at room temperature for 48 h gave compounds **12** and **13** (Scheme 4).

The ligand and the complexes were obtained as dark green, black and green powders respectively, air stable and soluble in DMSO and acetone. The elemental analysis (C, H, N, and S) and melting points data of these compounds are recorded in Table 1.

**Table 1:** Analytical and physical data for the ligand and complexes

Compounds	Colors	mp (^∘^C)	Calculated (found)			
			%C	%H	%N	%S
**11**	Dark green	296–298	59.68 (59.70)	3.69 (3.68)	7.33 (7.31)	8.38 (8.37)
**12**	Black	288–290	51.95 (51.98)	2.75 (2.78)	6.38 (6.36)	7.30 (7.27)
**13**	Green	214–216	51.95 (51.92)	2.75 (2.77)	6.38 (6.41)	7.30 (7.33)

**Fig. 1:**
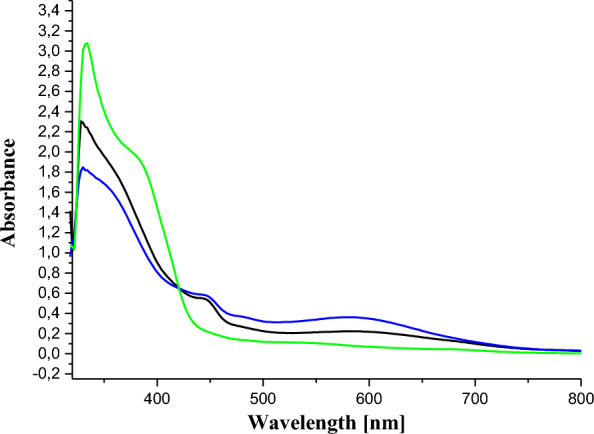
Electronic spectra of ligand **11** (black) and cobalt(II) complexes **12** (blue) and **13** (green)

The UV–VIS spectrum of ligand **11** showed a strong band in the ultraviolet range at 332 nm and moderate bands above 350 nm, attributed to the π → π* and n → π* transitions (due to the azo bridge), respectively (Fig. 1). The maximum absorption peak of azo compounds in general is around 330 nm in the UV–visible absorption spectrum due to the π → π* electronic transition of *trans* isomers [48]. In the context of this study, these absorption maxima observed in the ultraviolet region of the UV–Vis spectra of ligand **11** and of the synthesized complexes, **12** and **13**, are found at 332 nm, close to that reported in the literature. Furthermore, the electronic spectral data were very useful for the assignments of the stereochemistry of the metal complexes based on the positions and number of d → d transition peaks.

For the cobalt (II) complexes, the only possible configurations found in the literature are square-plane, tetrahedral and octahedral. Therefore, for complex of cobalt(II) hexadentate **12**, the only possible geometry that could be envisaged is octahedral. In fact, its electronic spectrum shows two bands of low intensities in the visible range. The first at 490 nm is attributed to the ^4^T1g(F) → ^4^A2g(F) transition and the second around 600 nm is due to the ^4^T1g(F) → ^4^T1g(P) transition [49]. These 2 absorptions are characteristic of an octahedral environment around the cobalt(II) ion complexes [50].

For the tetradentate complex **13**, a square-planar or tetrahedral configuration could be envisaged. Based on its electronic spectrum, it was possible to differentiate between these two alternative configurations as follows. The absence of absorptions between 600 and 700 nm which are characteristic for tetrahedral cobalt(II) complexes [51, 52], ruled out the hypothesis of a tetrahedral geometry for **13**. Moreover, the presence of an absorption (of very low intensity) above 500 nm (Fig. 1) makes more plausible the hypothesis of a low-spin square-planar geometry for this complex [53, 54]. As a consequence, on the basis of the LCAO approach, the central Co(II) ions should be hybridized sp^3^d^2^ and dsp^2^ respectively to comply with the octahedral and square-planar geometries of the coordination spheres in compounds **12** and **13** respectively. The absorption spectra of the ligand and complexes are represented in Fig. 1.

**Fig. 2:**
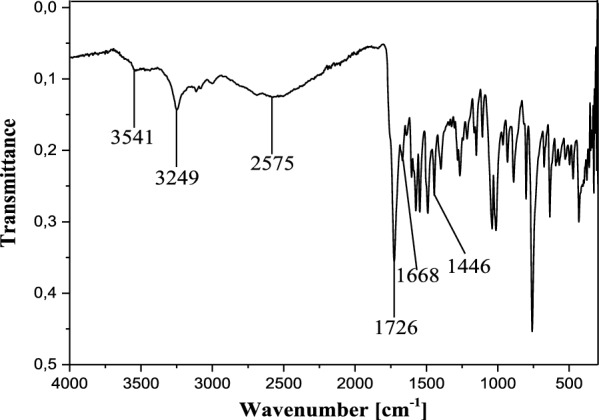
Infrared spectrum of compound **11**

**Fig. 3:**
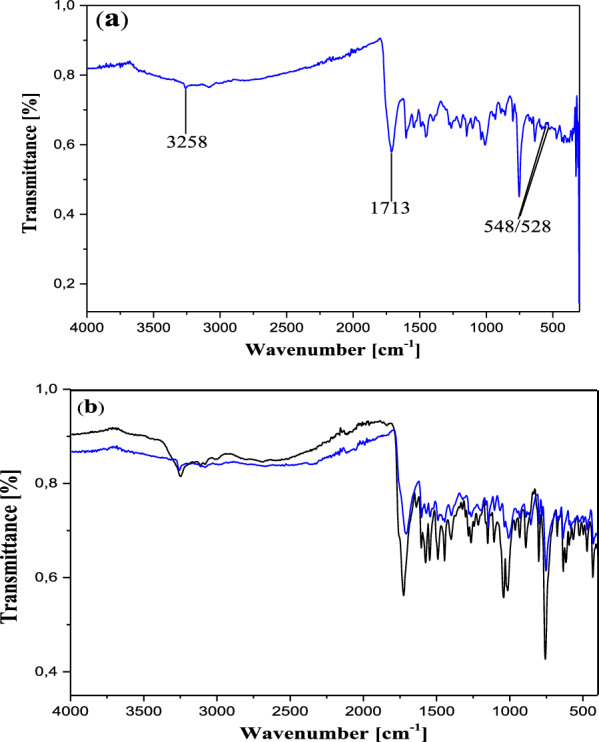
**a** Infrared spectrum of compound **12**. **b** Superposed infrared spectra of compounds **11** (black) and **12** (blue)

**Fig. 4:**
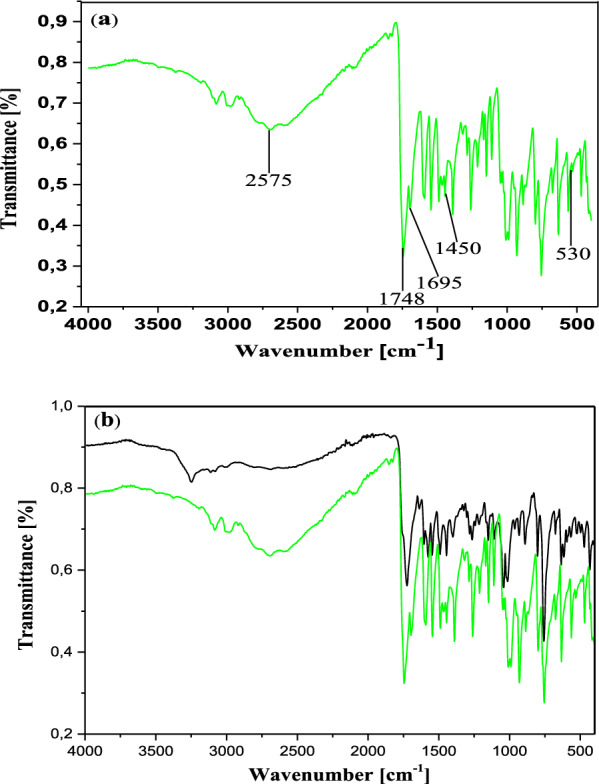
**a** Infrared spectrum of compound **13. b** Superposed infrared spectra of compounds **11** (black) and **13** (green)

In the IR spectrum, the free ligand C_19_H_14_N_2_O_5_S shows a very strong and sharp band with well-structured peaks at 1726 and 1668 cm^−1^ due to the ν(C=O) (ketone and acid respectively) present in the molecule (Fig. 2). In the IR spectra of the complexes [Co_2_(C_19_H_12_N_2_O_5_S)_2_] (Figs. 3a and 4a), these bands appear but with a pronounced shift towards higher frequencies at 1748 cm^−1^ and 1695 cm^−1^ respectively (for **13**), and towards lower frequencies around 1713 cm^−1^ (for **12**), indicating the involvement of the corresponding oxygen in the coordination with the central Co^2+^ ion. In these complexes, the values of ν(N=N) observed at 1446 cm^−1^ in the ligand remain constant, meaning that the azo function does not participate in the coordination. The other atoms involved in the coordination bonds in these molecules are the oxygen atoms of the two phenolic hydroxyl groups and that of the carboxylic acid function present in ligand **11**. The absence of the ν(OH) frequencies in the IR spectrum of the complex **13**, observed at 3541 cm^−1^ and 3248 cm^−1^ (Fig. 4b) in the starting ligand and assigned to free (2'-OH) and chelated (4''-OH) phenolic hydroxyls respectively, suggests the participation of the corresponding OH groups in the coordination with deprotonation (Fig. 4a). In the IR spectrum of the complex **12**, the signal of the hydroxyl (2'-OH) appears in the higher frequency region at 3258 cm^−1^ and one can notice the disappearance of the signal of the carboxylic acid hydroxyl around 2575 cm^−1^. These observations are suggestive of the participation of the corresponding oxygen atoms in the coordination without and with deprotonation respectively. The new bands that appeared in the IR spectrum of the complex in the region 521–570 cm^−1^ at 548 and 528 cm^−1^ (complex **12**) and at 530 cm^−1^ (complex **13**) were attributed to the Co–O bonds [55, 56] between the central cobalt ion and all the oxygen atoms involved in coordination. The relative intensities as well as the provisional assignments of the various bands mentioned above are given in the Table 2.

**Table 2:** Infrared spectral data for the ligand and complexes

Compounds	Infrared spectral data (cm^−1^)						
	ν (2′-OH)	ν (4′-OH)	ν (OH)acid	ν (C=O)ketone	ν (C=O)acid	ν (N=N)	ν (Co–O)
**11**	3541	3249	2575	1726	1668	1446	–
**12**	–	3258	–	1713	1713	1446	528/548
**13**	–	–	2577	1748	1695	1450	530

**Fig. 5:**
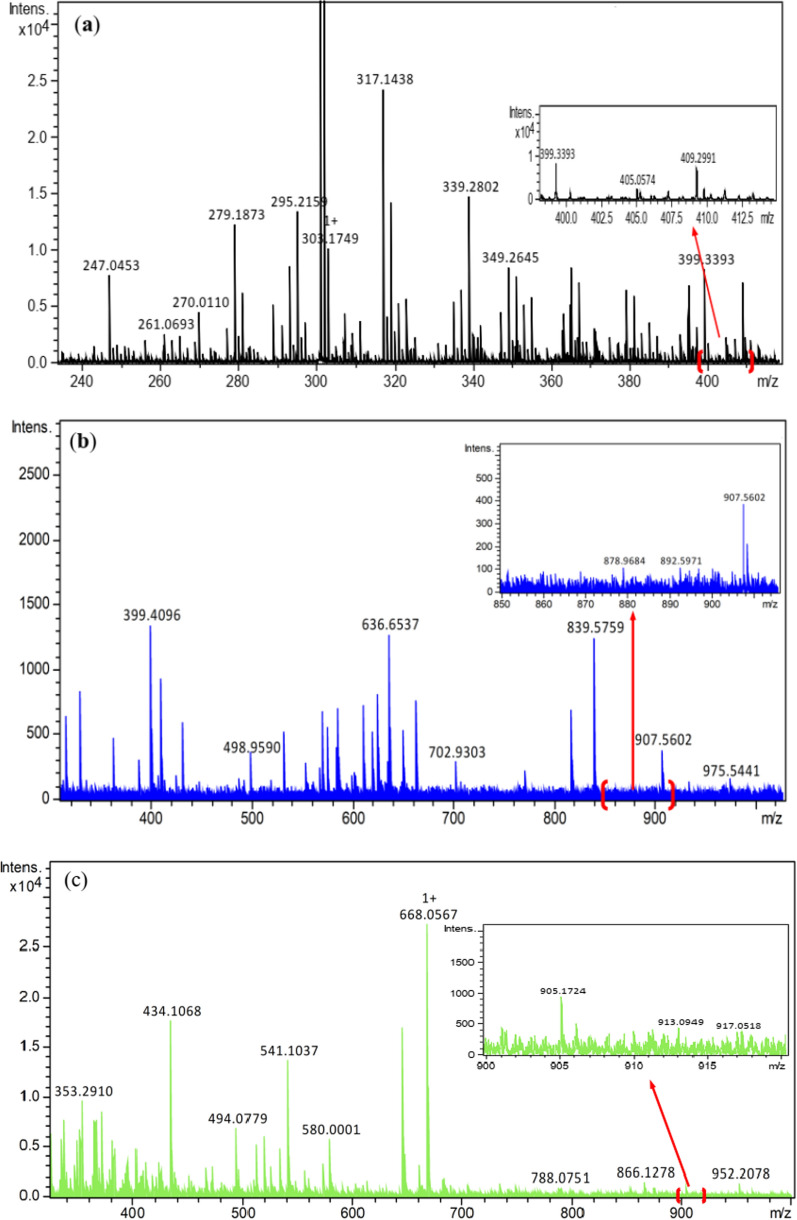
**a** HRESI + mass spectrum of azo ligand **11**. **b** HRESI + mass spectrum of complex **12**. **c** HRESI + mass spectrum of complex **13**

**Scheme 5:**
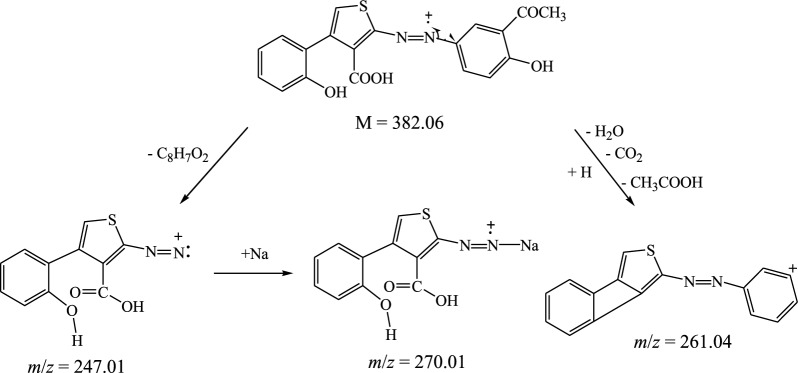
Suggested fragmentation pattern of azo ligand **11**

**Scheme 6:**
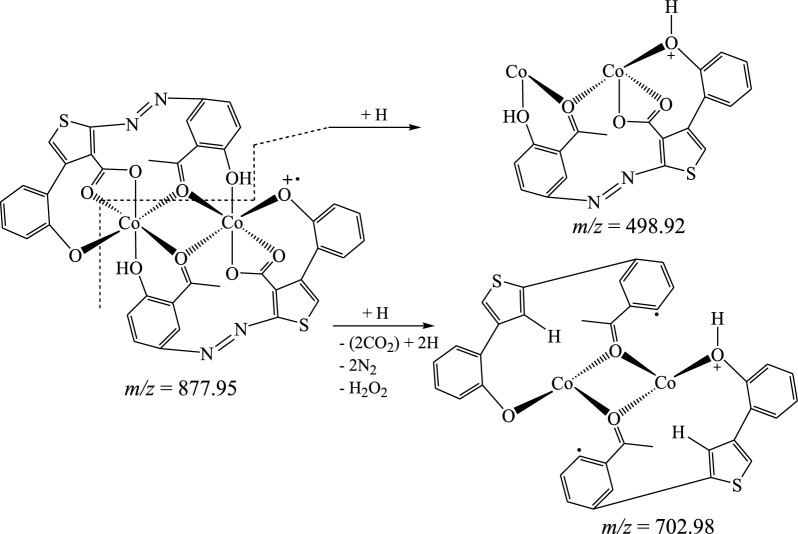
Suggested fragmentation pattern of Co(II) complex **12**

**Scheme 7:**
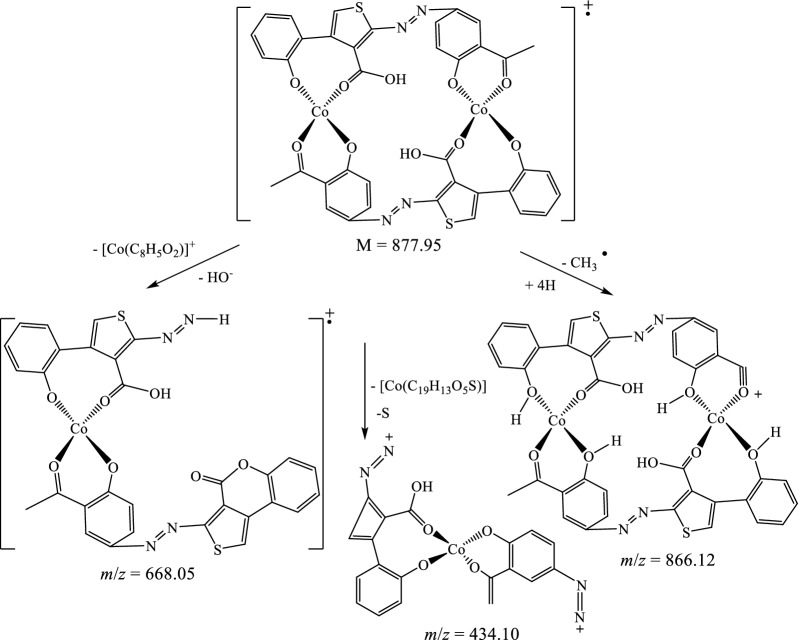
Suggested mass fragmentation pattern of Co(II) complex **13**

The suggested structures were supported by the mass spectral data of the free azo dye ligand and its Co(II) complexes, which were compatible with the molecular ion fragments (Fig. 5). Some of the fragments observed in the mass spectra of the ligand **11** and its Co(II) complexes **12** and **13** are rationalized in the fragmentation Schemes 5, 6 and 7.

**Fig. 6:**
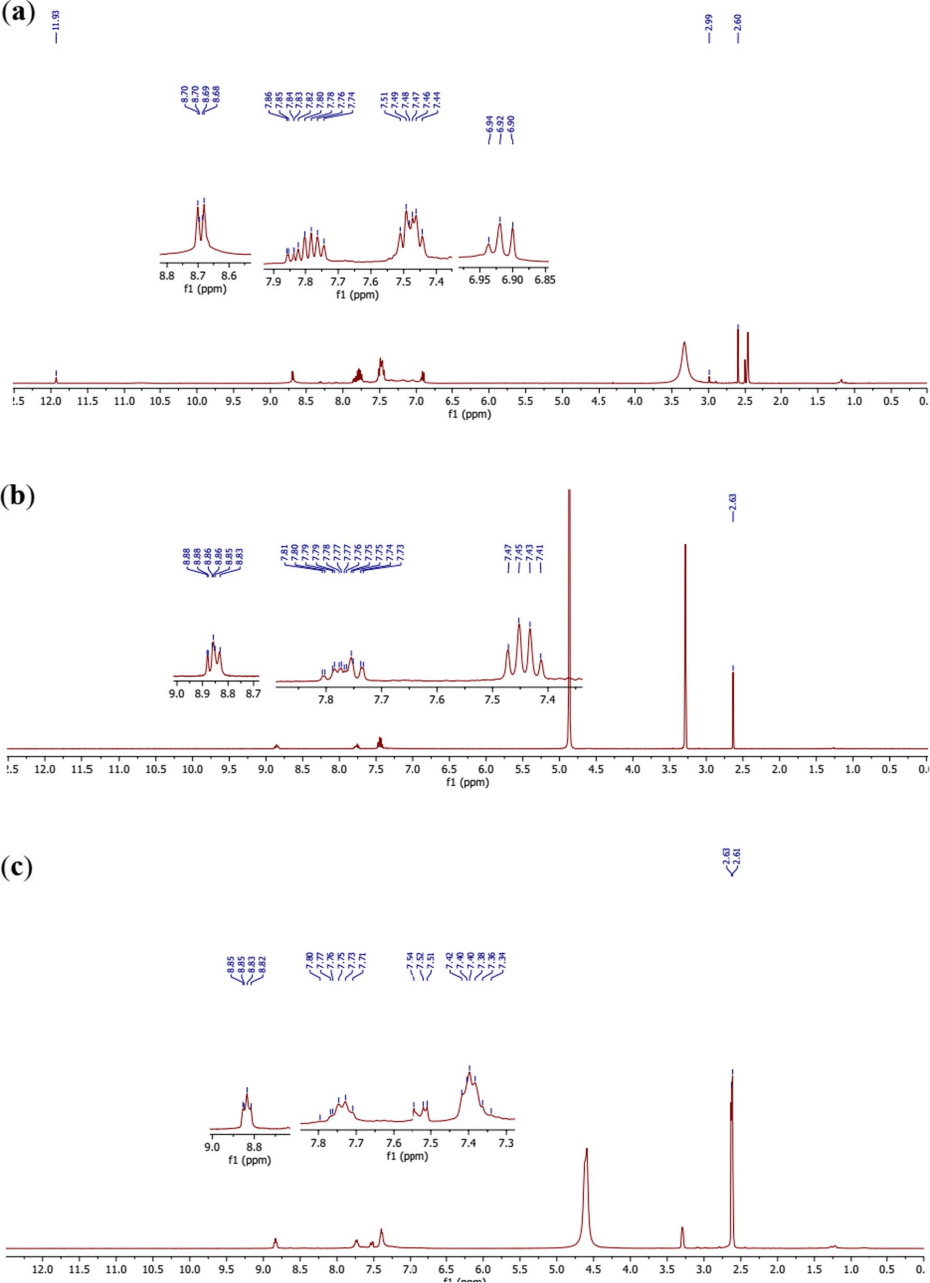
**a** ^1^H-NMR spectrum of the ligand **11**. **b** 1H-NMR spectrum of the complex **12. c**.^1^H-NMR spectrum of the complex **13**

Comparative ^1^H NMR spectra of the ligand (Fig. 6a) and the complexes (Fig. 6b, c) clearly show that the ligand undergoes deprotonation with complexation. Indeed, in the spectra of complexes **12** (Fig. 6b) and **13** (Fig. 6c), the 2′-OH at 2.99 ppm in the ligand was not seen in Fig. 6b, whereas, the 2′-OH at 2.99 ppm and 4″-OH at 11.93 ppm in the ligand were not seen in Fig. 6c. These observations confirm the formation of the Co–O bonds with the corresponding oxygen atoms. Moreover the same signals with almost the same multiplicities are observed in the spectra of the ligand and the complexes with respect to the aromatic protons.

**Fig. 7:**
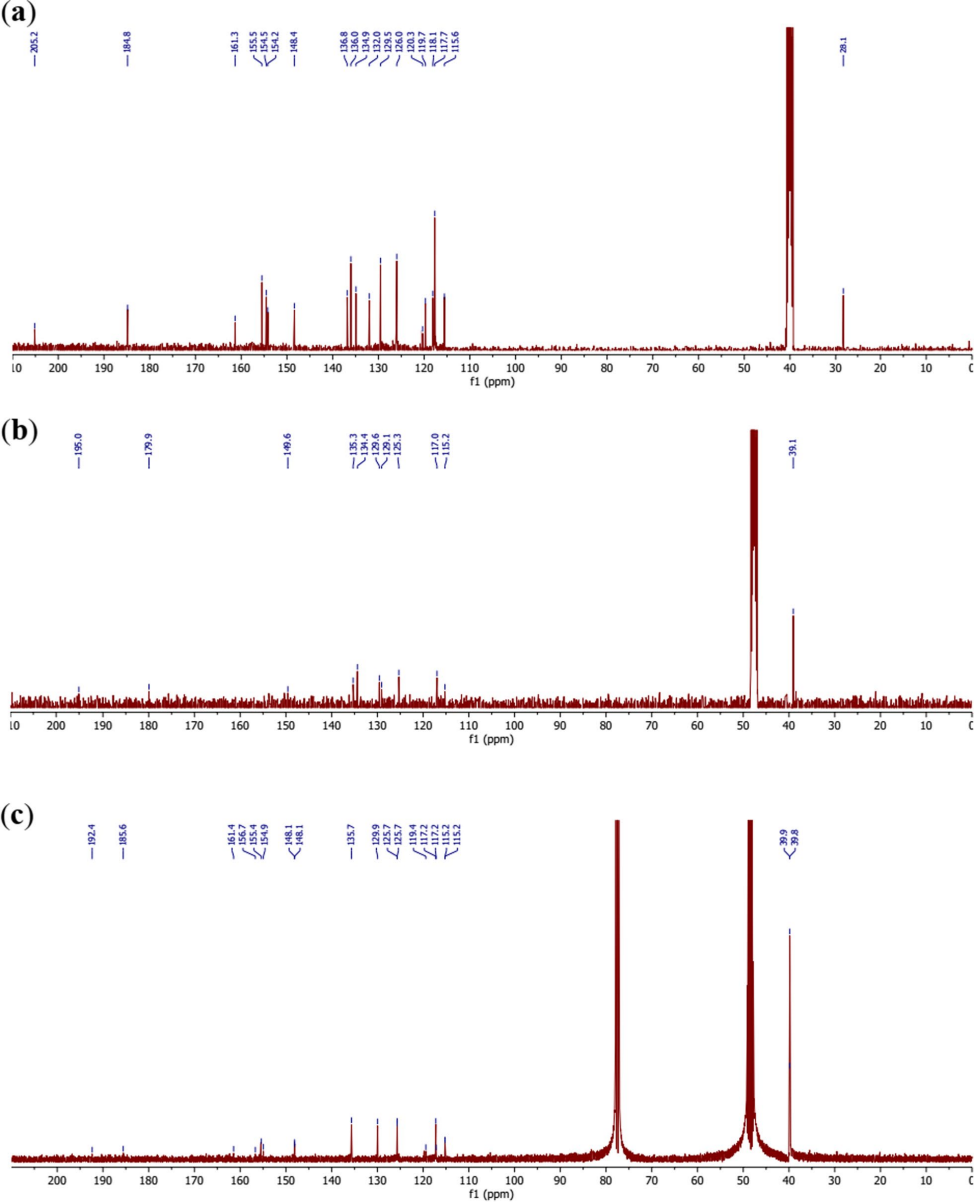
**a** ^13^C-NMR spectrum of the ligand **11. b** ^13^C-NMR spectrum of the complex **12. c**.^13^C-NMR spectrum of the complex **13**

The ^13^C NMR spectrum of the ligand **11** (C_19_H_14_N_2_O_5_S) (Fig. 7a) displays 19 signals due to the 19 carbon atoms present in this molecule. The most important being the carbon atoms bearing the coordinating oxygen atoms, found at 205.2 ppm, 184.8 ppm, 161.3 ppm and 155.5 ppm for the carbons 3″–**CO**CH_3_, 3–**CO**OH, C-4″ and C-2′, respectively.

Thus, the comparison of this spectrum with those of the [Co_2_(C_19_H_12_N_2_O_5_S)_2_] complexes **12** and **13** (Fig. 7b, c) made it possible to assign the carbonyls 3″-**CO**CH_3_ and 3-**CO**OH the chemical shift values 195.0 ppm and 179.9 ppm, respectively, and the phenolic carbons C-4″ and C-2′ the values 156.5 ppm and 156.0 ppm, respectively in the complex **12** whereas the values 192.4 ppm, 185.6 ppm, 161.4 ppm and 156.7 ppm could comparatively be assigned in the complex **13**, respectively for the above-mentioned atoms. The chemical shifts of the ligand and the complexes are summarized in Table 3.

**Table 3:** ^13^C and ^1^H NMR (DMSO-*d*_6_) data of the ligand **11** and those of the complexes **12** and** 13**

Positions	Ligand 11		Complex 12		Complex 13	
	*δ* ^13^_C_ in ppm	*δ* ^1^_H_ in ppm	*δ* ^13^_C_ in ppm	*δ* ^1^_H_ in ppm	*δ* ^13^_C_ in ppm	*δ* ^1^_H_ in ppm
2	154.5	–	–	–	155.4	–
3	154.2	–	–	–	154.9	–
3-COOH	184.8	–	179.9	–	185.6	–
4	148,4	–	149.6	–	148.1	–
5	119.7	6.94	129.1	8.83	–	7.54
1′	115.6	–	115.2	–	115.2 × 2	–
2′	155.5	–	156.0	–	156.7	–
2′-OH	–	2.99	–	–	–	–
3′	117.7	7.46–7.49	117.0	7.41–7.47	117.2 × 2	7.34–7.42
4′	136,0	7.74–7.85	135.3	7.73–7.81	135.7	7.71–7.80
5′	126.0	7.46–7.49	125.3	7.41–7.47	125.7 × 2	7.34–7.42
6′	129.5	8.69	129.6	8.87	129.9	8.83
1″	134.9	–	134.4	–	–	–
2″	136,8	7.74–7.85	–	7.73–7.81		7.71–7.80
3″	120.3	–	–	–	119.4	–
3″-COCH_3_	205.1	–	195.0	–	192.4	–
3″-COCH_3_	28.1	2.60	39.1	2,63	39.9 39.8	2.63 2.61
4″	161.3	–	156.5	–	161.4	–
4″-OH	–	11.93	–	–	–	–
5″	118.1	6.91	–	7.41–7.47	–	7.51
6″	132.0	7.74–7.85	–	7.73–7.81	–	7.71–7.80

**Fig. 8:**
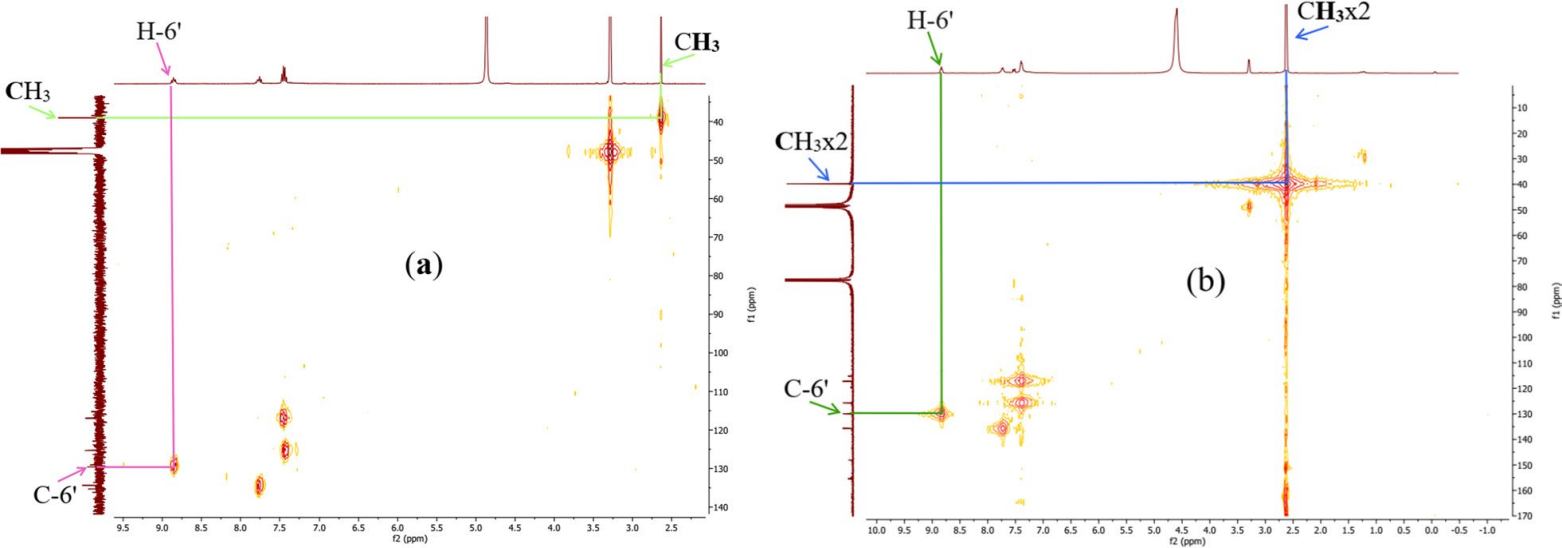
HSQC spectra of complexes **12** (**a**) and **13** (**b**) with some correlations

Figure 8 summarizes the two most significant interactions that were seen in the HSQC spectra of the ligand and compounds **12** and **13**. The first of these is the correlation spots between the methyl protons at 2.63 ppm (in **11**) (one signal), and at 2.63 and 2.61 ppm (in **13**) (two signals) and their carbons at 39.1 ppm and at 39.9 and 39.8 ppm respectively. On the other hand, correlation spots between protons H-6' at 8.87 ppm (in **12**) and at 8.83 ppm (in **13**) and their carbons at 129.6 ppm (in **12**) and at 129.9 ppm (in **13**) indicate the presence of the acetophenone fragment and that of the thiophenic moieties on each side of the N = N bridge.

**Fig. 9:**
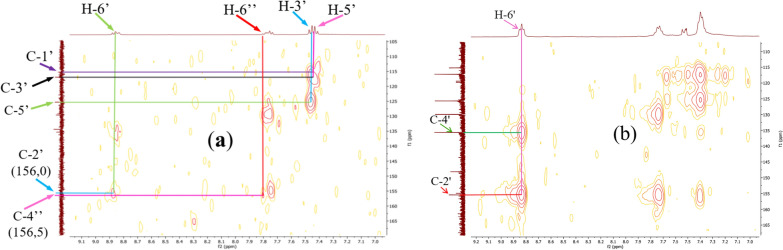
HMBC spectra of complexes **12** (**a**) and **13** (**b**) with some correlations

The long-distance couplings (^2^* J* and ^3^* J*) between the protons and the carbons of the chelating ligand moieties were highlighted by the HMBC experiment (Fig. 9). Indeed, it allowed to reconstruct the carbon skeleton of the coupling fragment through correlation spots between the 4″-OH proton (11.93 ppm) and the C-4″ carbons (161.3 ppm) and C-5″ (118.1 ppm), the proton H-6″ (around 7.85 ppm) and the carbons C-2″ (136.8 ppm) and C-4″ (161.3 ppm), the H-5″ proton (6.91 ppm) and the C-3″ carbons (120.0 ppm) and finally between the methyl CH_3_ (2.60 ppm) and the carbonyl C=O (205.1 ppm), thus eliminating the hypothesis of multiple couplings on the aromatic ring of the coupler. Some of these correlations were also found in the HMBC spectra of the complexes despite their high complexity due to the overlapping of homologous proton systems of the chelating ligand moieties.

**Fig. 10:**
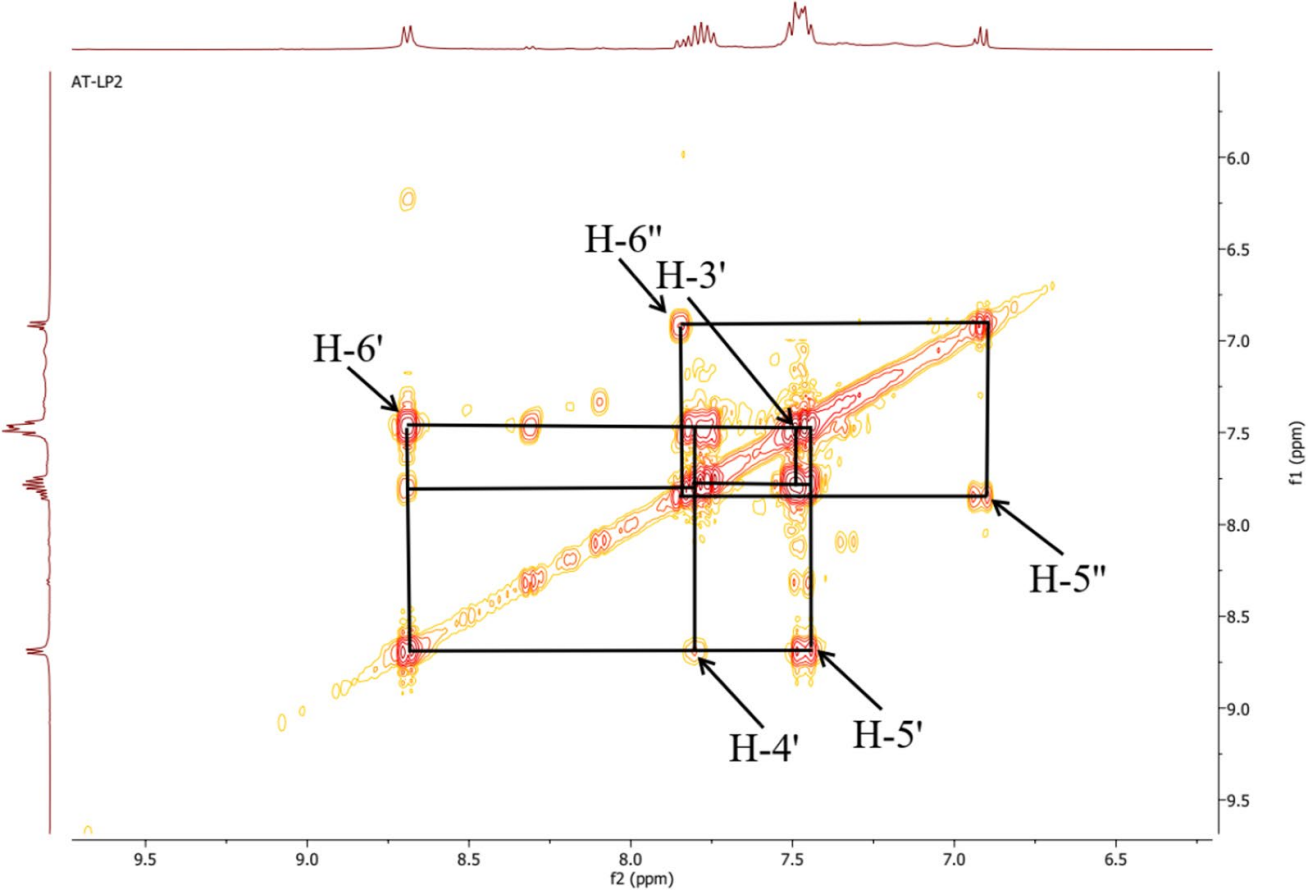
COSY ^1^H-^1^H spectrum of ligand **11** with some correlations

**Fig. 11:**
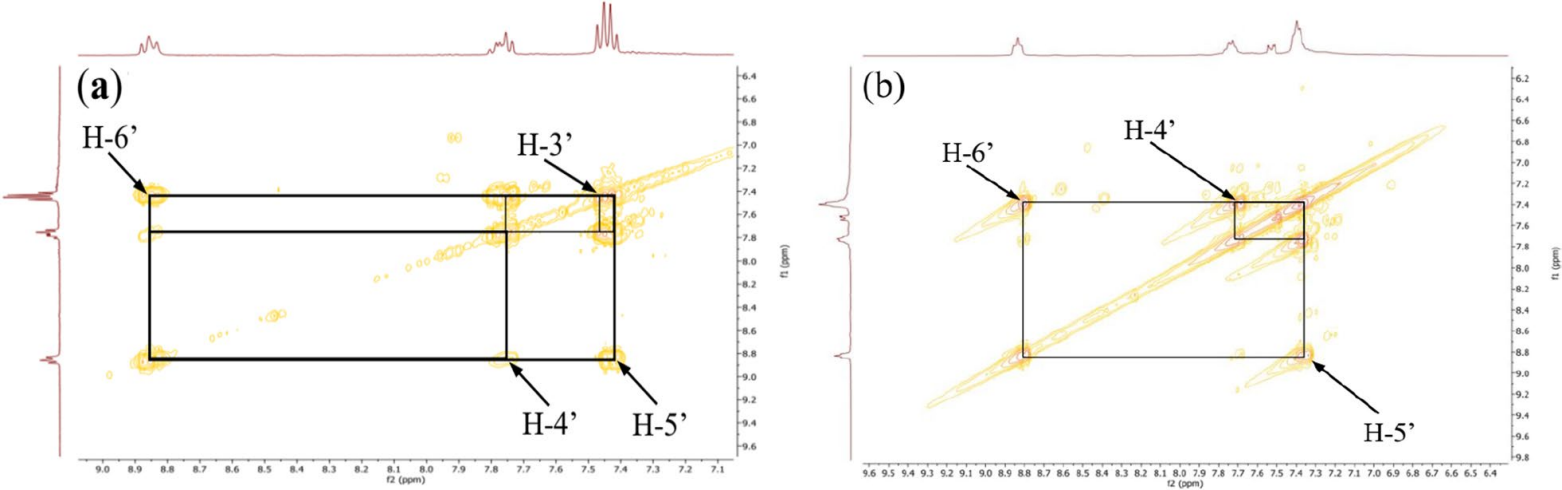
COSY ^1^H-^1^H spectra of complexes **12** (**a**) and **13** (**b**) with some correlations

The COSY ^1^H-^1^H experiment of the ligand (Fig. 10) clearly showed the correlation squares between the aromatic protons belonging to the molecular fragments on either side of the azo bridge. For the complexes, the most visible correlations are those of the benzene ring for the above mentioned similar reasons (Fig. 11).

**Fig. 12:**
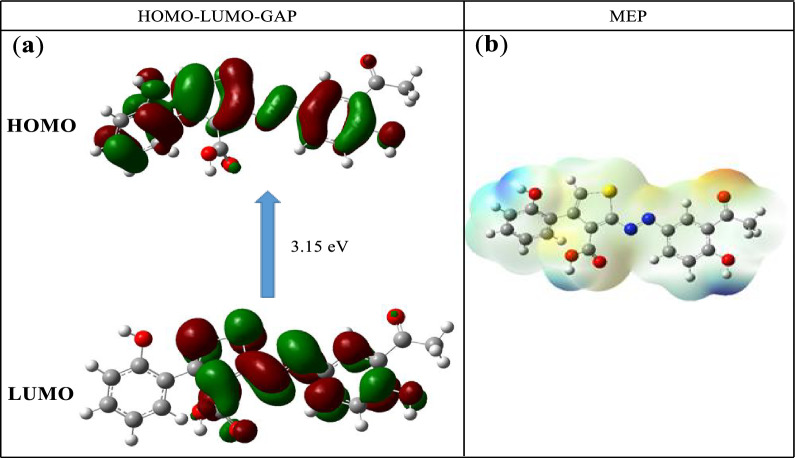
Structures of the FMO (HOMO and LUMO) (**a**) and MEP (**b**) of compound **11**

Theoretical calculations were performed on the ligand to determine the most reactive sites of the unsaturated system. The energies and electronic densities of the frontier molecular orbitals (FMO), HOMO and LUMO, as well as the molecular electrostatic potential (MEP) are important electronic parameters for this purpose [57, 58]. The structures of the FMO and the MEP obtained from a B3LYP/6-311G mode of calculations are given in Fig. 12. The E_HOMO_ and E_LUMO_ values are—6.114 eV and—2.960 eV respectively, resulting in an energy gap of 3.15 eV.

### XDR analysis

**Fig. 13:**
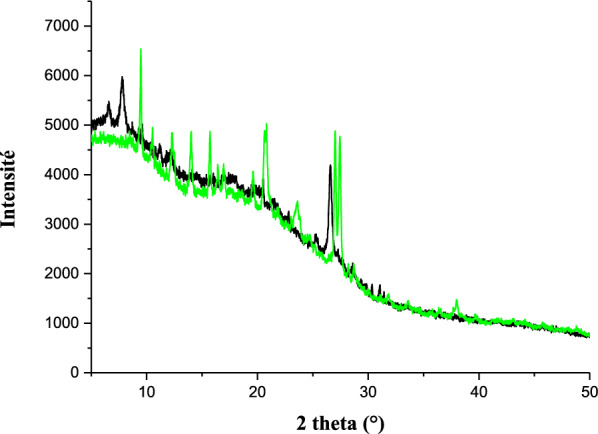
Ex situ PXRD pattern (Cu Kα1 radiation) of XRD of compounds **11** (black) and **13** (green)

**Fig. 14:**
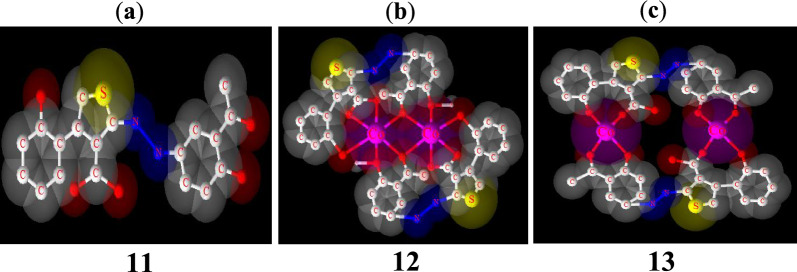
Optimized 3D view of compounds **11, 12** and **13**. The structures were drawn with the program ACD/3D viewer (freeware) of ACD/Labs

The powder X-ray diffraction of ligand **11** and complex **13** are different from each other (Fig. 13) and indicates a good crystalline structure and a good purity of these compounds. The spectra of compound **13** shows a significant number of sharp bands or peaks. This suggests that it is made up of well-organized particles. All the new peaks exhibited in the diffractogram of the complex **13** are in agreement with the fact that it is different from the ligand **11**. The optimized 3D view of compound **11, 12** and **13** are clearly presented in Fig. 14.

### Biology

#### Antibacterial activity

The comparative study of the activity of the starting 2-aminothiophen (**7)** and the tree new compounds (**11**, **12** and **13**) was carried out on bacteria strains such as *Staphylococcus aureus* ATCC25923, *Pseudomonas aeruginosa*, *Escherichia coli* ATCC25922 and *Klebsiella pneumoniae* 22. Screening results showed that compound **7** had a moderate activity (CMI = 128 µg/mL) and (CMI = 64 µg/mL) on *Escherichia coli* ATCC25922 and *Klebsiella pneumoniae* 22 respectively, but its highest activity (CMI = 32 µg/mL) was found on *Pseudomonas aeruginosa* and *Staphylococcus aureus* ATCC25923 strains. These activities decrease in the azoic ligand **11** on *Pseudomonas aeruginosa* (CMI = 64 µg/mL) and *Staphylococcus aureus* ATCC25923 (CMI = 128 µg/mL); increase on *Escherichia coli* ATCC25922 (CMI = 32 µg/mL) and remains constant on *Klebsiella pneumoniae* 22 (CMI = 64 µg/mL). Complex **12** had no activity on two strains *Staphylococcus aureus* ATCC25923 and *Escherichia coli* ATCC25922, but had a moderate activity (CMI = 128 µg/mL) and (CMI = 64 µg/mL) on *Pseudomonas aeruginosa* and *Klebsiella pneumoniae* 22 respectively, while complex **13** had no activity on all strains except on *Klebsiella pneumoniae* 22 (CMI = 64 µg/mL) where the activity remains constant with respect to precursor **7**. All data are summarized in Table 4.

**Table 4:** Antimicrobial activity (MIC and MBC in µg/mL) of synthesized compounds as well as reference antimicrobial drugs

Coumpounds	Inhibition parameters	*S. aureus* ATCC25923	*P. aeruginosa*	*E. coli* ATCC25922	*K. pneumoniae* 22
**7**	MIC MBC MBC/CMI	32 > 256 /	128 > 256 /	64 > 256 /	64 > 256 /
**11**	MIC MBC MBC/CMI	32 > 256 /	64 > 256 /	64 > 256 /	128 > 256 /
**12**	MIC MBC MBC/CMI	128 > 256 /	32 > 256 /	> 256 > 256 /	64 > 256 /
**13**	MIC MBC MBC/CMI	64 > 256 /	64 > 256 /	128 > 256 /	64 > 256 /
Reference drugs*	MIC MBC MBC/CMI	8 16 2	8 16 2	16 32 2	16 32 2

/ not determined, *MIC* Minimum Inhibitory Concentration, *MBC* Minimum Bactericidal Concentration

^*^Ciprofloxacin was tested together with compounds **7**, **11, 12** and **13**

#### Cytotoxic activity

To investigate the potential use of compounds **7**, **11, 12** and **13**, their cytotoxicity was evaluated. None of the tested samples showed hemolytic activities against red blood cells at concentrations up to 128 µg/mL (Table 5). However, at the highest concentration tested in this study (256 μg/mL), complexes caused less than 4% of hemolysis. This finding highlights that complexes are slightly hemolytic at 256 μg/mL.

**Table 5:** Cytotoxicity of compounds against red blood cells

Compounds	Cell lysis (%)						
	4 µg/mL	8 µg/mL	16 µg/mL	32 µg/mL	64 µg/mL	128 µg/mL	256 µg/mL
**7**	0	0	0	0	0	0	0
**11**	0	0	0	0	0	0	0
**12**	0	0	0	0	0	0	1,76 ± 0,000
**13**	0	0	0	0	0	0	2.44 ± 0.003

## Conclusion

In summary, two novel binuclear complexes of Co(II) with a novel multifunctional azo ligand incorporating a thiophenic and a phenolic moiety have been prepared, and their structures fully assigned on the basis of the available elemental, powder XRD and spectroscopic data. IR spectral data show that ligand **11** behaves as a hexadentate ligand in **12**, coordinating via all electron-donating oxygen atoms, and as a tetradentate ligand in **13**, coordinating via all oxygen atoms except that of carboxylic acid functions. It was established that in both complexes the central Co(II) ions were sp^3^d^2^ and dsp^2^ hybridized in **12** and **13,** respectively. The models of metal ion binding to the coordination sites of chelating ligands display octahedral and planar-square geometries in complexes **12** and **13** respectively in agreement with their UV–Vis data. From the biological screenings carried out on selected strains of multiresistant bacteria, it was found that compared to the free ligand, the coordination compounds have relatively very low activity on most of the tested strains. Nevertheless, further similar studies need to be carried out on a more large number of pathogens before a rational conclusion could be drawn on the structure–activity relationship linked with the coordination process.

## Materials and methods

### Instrumental method

All the reagents mentioned in this work were purchased from Aldrich and Fluka and were used without further purification. Melting points are corrected and were determined with a STUART SCIENTIFIC Melting Point Apparatus Model SMP3 at a heating rate of 2 °C/min. TLCs were performed on prefabricated silica gel plates, consisting of silica gel 60 F_254_ on aluminum foil with a fluorescent indicator. A mixture of ethyl acetate and hexane (1:1) was used as eluent to develop the TLC plates and the spots were visualized using iodine vapor or by spraying with 10% H_2_SO_4_ and heating at 100 ℃ for 2 min. The IR spectra were recorded with a Bruker Alpha spectrophotometer using the ATR (Attenuated Total Reflectance) technique on a diamond crystal. The HRESI-MS spectra were recorded on a Compact BRUKER brand spectrometer with a DIONEX Ultimate 3000 brand LC chain. Nuclear magnetic resonance (NMR) experiments (1D and 2D) were performed in DMSO-*d*_6_ and MeOH-*d*_4_/CCl_4_ on a 400 MHz JEOL ECZ spectrophotometer equipped with 5-mm digital auto tune Royal probe (JEOL USA, Peabody, MA). ^1^H-NMR spectral data were recorded at 400 MHz, while ^13^C-NMR data were measured at 100 MHz both with TMS used as internal reference. Powder XRD data was collected on a STOE Stadi-p X-ray powder diffractometer (STOE & Cie GmbH, Darmstadt, Germany) with Cu K_α1_ radiation (λ = 1.54056 Å; Ge monochromator; flat samples) in transmission geometry with a DECTRIS® MYTHEN 1 K detector (DECTRIS, Baden-Daettwil, Switzerland). Elemental analyses were performed with a Euro Vector CHNS-O element analyzer (Euro EA 3000) or a vario MICRO Cube (Co. Elementar Analysen Systeme). Theorical calculations were performed with Gaussian 9 software in a B3LYP/6-311G mode.

### Synthesis of 2-[(E)-(3-acetyl-4-hydroxyphenyl)diazenyl]-4-(2-hydroxyphenyl)thio-phene-3-carboxylic acid (11)

To the thienyldiazonium ion in solution, 1.35 g (9.93 mmol) of **10** was added dropwise with stirring over 30 min and the mixture was further stirred for an additional 30 min to complete the reaction. At the end of this, 5 mL of a potassium bicarbonate solution (10% KHCO_3_) was added in small portions to the mixture to neutralize the excess acid. 10 min later, a volume of 50 mL of ice water was added to the mixture and the latter was left to stand for 24 h before being filtered. The product obtained is then washed cold and then hot with water to remove any impurities in order to give 1.97 g of **11** (from 2 g of **7**) as dark green powder; R_f_: 0.6, mp: 296–298 °C, yield 55.97%; HRESI-MS: 405.0574 (M + Na, 0.54%). UV–Vis: λ_max_ (acetone): 332, 445, 586 nm. IR (ATR): 1450 cm^−1^ (N=N), 1667 (C=O)_acid_, 1729 (C=O)_ketone_, 1166 (C–O), 3541 (2′-OH), 3248 (4″-OH), 2579 (3-CO**OH**) cm^−1^. ^1^H NMR (DMSO-*d*_6_) *δ* ppm: 8.69 (dd, 1H, J = 8.1 and 1.2 Hz, H-6′), 7.80 (m, 1H, H-4′), 7.46 (m, 1H, H-5’), 7.21 (d, 1H; J = 7.1 Hz, H-3′), 6.94 (s,1H, H-5), 7.74 (d,1H, J = 1.9 Hz; H-2″), 6.91 (d, 1H, J = 8.1 Hz; H-5″), 7.85 (dd, 1H, J = 8.1 and 1.9 Hz; H-6″). ^13^C NMR (DMSO-*d*_6_) *δ* ppm: 154.5 (C-2), 154.2 (C-3), 184.8 (3-**CO**OH), 148.4 (C-4), 119.7 (C-5), 115.6 (C-1′), 155.5 (C-2′), 117.7 (C-3′), 136.0 (C-4′), 126.0 (C-5′), 129.5 (C-6′), 134.9 (C-1″), 136.8 (C-2″), 120.3 (C-3″), 205.1 (3″-**CO**CH_3_), 161.3 (C-4″), 118.1 (C-5″) and 132.0 (C-6″). Anal. Calcd. for C_19_H_14_N_2_O_5_S (382.0623): C, 59.68; H, 3.69; N, 7.33; S, 8.38; found: C, 59.70; H, 3.68; N, 7.31; S, 8.37.

### Synthesis of complexes 12 and 13 ([Co_2_(C_19_H_12_N_2_O_5_S)_2_])

To a magnetically stirred solution of the ligand **11** (300 mg; 0.79 mmol) in DMSO (4 mL) a solution of Co(C_2_O_4_)‧2H_2_O (140 mg; 0.77 mmol) in 3 mL EtOH/MeOH (2:1) was gradually added and the reaction volume made up to 20 mL with ethanol. After 48 h, the product formed was collected by simple filtration then washed with ethanol after 10 days to give 53 mg of **12** as a black precipitate. From the resulted filtrate, 45 mg of **13** was collected after 30 days as a green precipitate. After washing, the complexes were left to stand and the solvent evaporated after 24 h. Compound **12**: R_f_: 0.66, mp: 288–290 °C, yield 31.18%; HRESI-MS: 878.9684 (M + H, 0.10%). UV–Vis: λ_max_ (acetone): 332, 436, 490, 590 nm. IR (ATR): 3258 cm^−1^ (OH)_chelated_, 1713 (C=O_ketone_), 1713 (C=O_acid_), 1446 (N=N), 528/548 (Co–O) cm^−1^. ^1^H NMR (MeOH-*d*_4_/CHCl_3_-*d*_1_) *δ* ppm: 8.83 (s, 2H, H-5), 7.41–7.47 (m, 2H, H-3′), 7.73–7.81 (m, 2H, H-4′), 7.41–7.47 (m, 2H, H-5′), 8.87 (m, 2H, H-6′), 7.73–7.81 (m, 2H, H-2″), 7.41–7.47 (m, 2H, H-5’’), 7.73–7.81 (m, 2H, H-6’’). ^13^C NMR (DMSO-*d*_6_) *δ* ppm: 179.9 (3-**CO**OH), 148.4 (C-4), 129.1 (C-5), 115.2 (C-1′), 156.0 (C-2′), 117.0 (C-3′), 135.3 (C-4′), 125.3 (C-5′), 129.6 (C-6′), 134.4 (C-1’’), 195.0 (3’’-**CO**CH_3_) and 156.5 (C-4’’). Anal. Calcd. for [Co_2_(C_19_H_12_N_2_O_5_S)_2_] (877.9592): C, 51.95; H, 2.75; N, 6.38; S, 7.30; found: C, 51.98; H, 2.78; N, 6.36; S, 7.27. Compound **13**: R_f_: 0.63, mp: 214–216 °C, yield 26.47%; HRESI-MS: 917.0518 (M + K, 0.18%). UV–Vis: λ_max_ (acetone): 332, 382, 538 nm. IR (ATR): 2573 cm^−1^ (OH_acid_), 1748 (C=O_ketone_), 1695 (C=O_acid_), 1446 (N=N), 530 (Co–O) cm^−1^. ^1^H-NMR (MeOH-*d*_4_/CHCl_3_-*d*_1_) *δ* ppm: 7.54 (s, 2H, H-5), 7.34–7.42 (m, 2H, H-3′), 7.71–7.80 (m, 2H, H-4′), 7.34–7.42 (m, 2H, H-5′), 8.83 (m, 2H, H-6′), 7.51 (m, 2H, H-5″), 7.71–7.80 (m, 2H, H-6″). ^13^C NMR (MeOH-*d*_4_/CHCl_3_-*d*_1_) *δ* ppm: 155.4 (C-2), 154.9 (C-3), 185.6 (3-**CO**OH), 148.1 (C-4), 115.2 (C-1’), 156.7 (C-2’), 117.2 (C-3′), 135.7 (C-4′), 125.7 (C-5′), 129.9 (C-6′), 192.4 (3″-**CO**CH_3_) and 161.4 (C-4″). Anal. Calcd. for [Co_2_(C_19_H_12_N_2_O_5_S)_2_] (877.9598): C, 51.95; H, 2.75; N, 6.38; S, 7.30; found: C, 51.92; H, (2.77); N, 6.41; S, 7.33.

### Antimicrobial evaluation

#### Tested microorganisms

Against four different bacterial species, the antibacterial activity was conducted. One Gram-positive *Staphylococcus aureus* ATCC25923 and three Gram-negative *Pseudomonas aeruginosa*, *Escherichia coli ATCC25922*, and *Klebsiella pneumoniae* 22 were the chosen bacteria. These microorganisms were collected from our laboratory collection. The different bacterial species were maintained at + 4 °C and activated on BBL^®^ nutrient agar (NA, Conda, Madrid, Spain) for 24 h before any antibacterial test.

##### Determination of minimum inhibitory concentration (MIC) and minimum microbicidal concentration (MMC)

The MICs were determined by the method of microdilution in a liquid medium [59]. Stock solutions of samples were prepared in an aqueous solution of Dimethyl Sulphoxide 10% (DMSO, Fisher Chemicals, Strasbourg, France) at a concentration of 512 µg/mL. From these stock solutions, successive dilutions in series of 2 were carried out in Mueller–Hinton broth (MHB). For each test, the sterility test (aqueous solution of DMSO at 10% + culture medium), the negative control (aqueous solution of DMSO at 10% + culture medium + inoculum) and the positive control (aqueous solution of DMSO at 10% + culture medium + inoculum + reference drug) were included. 100 μL of each concentration were introduced into a well of a 96-well (200 μL per well) microtiter plate containing 90 μL of MHB and 10 μL of inoculum were added to obtain a range of concentrations varying from 256 to 0.125 μg/mL. Plates were covered and incubated at 37 °C for 24 h on a shaker (Flow Laboratories) at 300 rpm. At the end of the various incubation times, the minimum inhibitory concentrations (MIC) were considered to be the lowest concentrations of substances for which we did not have any macroscopic growth materialized by the cloudy appearance of the well. Minimum bactericidal concentrations (MBCs) were determined by subculturing 10 μL (using 90 mm Petrie dishes) of the contents of wells where growth was not visible to the naked eye with Mueller–Hinton Agar (MHA) medium. The MBCs were defined as the lowest concentration that produced no growth following subculturing. Each test was run three times.

##### Cytotoxicity assay

The animals were bred in the animal house of the University of Dschang, Cameroon. The study was conducted according to the ethical guidelines of the Committee for Control and Supervision of Experiments on Animals (Registration number 173/CPCSEA, issued January 28, 2000), Government of India, on the use of animals for scientific research. Euthanasia was done using noninhaled agents. Hence, all the rats were anaesthesized via intraperitoneal injection of the mixture of ketamine (50 mg/ kg body weight, BW) and xylazine (10 mg/kg BW), in a dose that is commonly used for operation purposes. A conical tube containing EDTA as an anticoagulant was used to collect 10 mL of whole blood from albino rats using a heart puncture. Centrifugation at room temperature for 10 min at 1000 × g was used to collect erythrocytes, which were then washed three times in PBS buffer [60]. The cytotoxicity was evaluated as previously reported [60]. Death was confirmed using a combination of criteria including lack of pulse, breathing, corneal reflex, response to firm toe pinch; inability to hear respiratory sounds and; graying of the mucous membranes and rigor mortis before disposal of any animal remains.

### Supplementary Information

**Additional file 1: Figure S1. **Powder X-ray diffractogram of compound **11**. **Figure S2**. UV-VIS spectrum of compound **11**. **Figure S3**. IR spectrum of compound **11** with some assignments. **Figure S4**. HRESI+ mass spectrum of compound **11**. **Figure S5**. ^1^H-NMR spectrum of compound **11**. **Figure S6**. ^13^C-NMR spectrum of compound **11**. **Figure S7**. HSQC spectrum of compound **11**. **Figure S8**. COSY ^1^H-^1^H spectrum of compound **11** with some correlations. **Figure S9**. HMBC spectrum of compound **11**. **Figure S10**. UV-VIS spectrum of complex **12**. **Figure S11**. IR spectrum of complex **12**. **Figure S12**. HRESI+ mass spectrum of complex **12**. **Figure S13**. ^1^H-NMR spectrum of complex **12**. **Figure S14**. ^13^C-NMR spectrum of complex **12**. **Figure S15**. HSQC spectrum of complex **12** with some correlations. **Figure S16**. COSY ^1^H-^1^H spectrum of complex **12**. **Figure S17**. HMBC spectrum of complex **12** with some correlations. **Figure S18**. Comparison of the UV-VIS spectra of compound **11** (black) and **12** (blue). **Figure S19**. Comparison of the IR spectra of compounds **11** (black) and **12** (blue). **Figure S20**. Comparison of the ^1^H-NMR spectra of compounds **11** (a) and **12** (b). **Figure S21**. Comparison of the ^13^C-NMR spectra of compounds **11** (a) and **12** (b). **Figure S22**. Powder X-ray diffractogram of compound **13**. **Figure S23**. UV-VIS spectrum of compound **13**. **Figure S24**. IR spectrum of compound **13**. **Figure S25**. HRESI+ mass spectrum of compound **13**. **Figure S26**. ^1^H-NMR spectrum of compound **13**. **Figure S27**. ^13^C-NMR spectrum of compound **13**. **Figure S28**. HSQC spectrum of compound **13** with some correlations. **Figure S29**. COSY ^1^H-^1^H spectrum of compound **13**. **Figure S30**. HMBC spectrum of compound **13** with some correlations. **Figure S31**. Comparison of the powder X-ray diffractograms of compounds **11** (black) and **13** (green). **Figure S32**. Comparison of the UV-VIS spectra of compound **11** (black) and **13** (green). **Figure S33**. Comparison of the IR spectra of compounds **11** (black) and **13** (green). **Figure S34**. Comparison of the ^1^H-NMR spectra of compound **11** (a) and **13** (b). **Figure S35**. Comparison of the ^13^C-NMR spectra of compound **11** (a) and **13** (b).

## Data Availability

All spectra for the compounds’ characterization are provided as Additional material. Similarly, the raw data for all biological evaluations are available from the corresponding author upon reasonable request.

## Abbreviations

UV–Vis: Ultra-Violet-visible
IR: Infrared
FTIR: Fourier-transform infrared spectroscopy
HRESI-MS: High resolution electrospray ionization‐mass spectrometry
^1^H NMR: Proton nuclear magnetic resonance
^13^C NMR: Carbon nuclear magnetic resonance
DFT: Density functional theory
LCAO: Linear combinations of atomic orbitals
µg: Microgram
FMO: Frontier molecular orbitals
HOMO: Highest occupied molecular orbital
LUMO: Lowest unoccupied molecular orbital
MEP: Molecular electrostatic potential
°C: Degree centigrade
h: Hour
g: Gram
mg: Milligram
L: Liter
mL: Milliliter
µL: Microliter
MIC: Minimum inhibitory concentration
MMC: Minimum microbicidal concentration
MBC: Minimum bactericidal concentration
DMSO: Dimethylsulfoxide
Fig: Figure
