# Supplementary material for: Synthesis, characterization, and antibacterial activity studies of two Co(II) complexes with 2-[(E)-(3-acetyl-4-hydroxyphenyl)diazenyl]-4-(2-hydroxyphenyl)thiophene-3-carboxylic acid as a ligand
Source: BMC Chem. 2024 Apr 16;18(1):75. doi: 10.1186/s13065-024-01179-2 (PMC11022391; doi:10.1186/s13065-024-01179-2)
Supplement: Supplementary file 1 — Additional file 1: Figure S1. Powder X-ray diffractogram of compound 11. Figure S2. UV-VIS spectrum of compound 11. Figure S3. IR spectrum of compound 11 with some assignments. Figure S4. HRESI+ mass spectrum of compound 11. Figure S5. 1H-NMR spectrum of compound 11. Figure S6. 13C-NMR spectrum of compound 11. Figure S7. HSQC spectrum of compound 11. Figure S8. COSY 1H-1H spectrum of compound 11 with some correlations. Figure S9. HMBC spectrum of compound 11. Figure S10. UV-VIS spectrum of complex 12. Figure S11. IR spectrum of complex 12. Figure S12. HRESI+ mass spectrum of complex 12. Figure S13. 1H-NMR spectrum of complex 12. Figure S14. 13C-NMR spectrum of complex 12. Figure S15. HSQC spectrum of complex 12 with some correlations. Figure S16. COSY 1H-1H spectrum of complex 12. Figure S17. HMBC spectrum of complex 12 with some correlations. Figure S18. Comparison of the UV-VIS spectra of compound 11 (black) and 12 (blue). Figure S19. Comparison of the IR spectra of compounds 11 (black) and 12 (blue). Figure S20. Comparison of the 1H-NMR spectra of compounds 11 (a) and 12 (b). Figure S21. Comparison of the 13C-NMR spectra of compounds 11 (a) and 12 (b). Figure S22. Powder X-ray diffractogram of compound 13. Figure S23. UV-VIS spectrum of compound 13. Figure S24. IR spectrum of compound 13. Figure S25. HRESI+ mass spectrum of compound 13. Figure S26. 1H-NMR spectrum of compound 13. Figure S27. 13C-NMR spectrum of compound 13. Figure S28. HSQC spectrum of compound 13 with some correlations. Figure S29. COSY 1H-1H spectrum of compound 13. Figure S30. HMBC spectrum of compound 13 with some correlations. Figure S31. Comparison of the powder X-ray diffractograms of compounds 11 (black) and 13 (green). Figure S32. Comparison of the UV-VIS spectra of compound 11 (black) and 13 (green). Figure S33. Comparison of the IR spectra of compounds 11 (black) and 13 (green). Figure S34. Comparison of the 1H-NMR spectra of compound 11 (a) and 13 (b). Figure S35. Comparison of the 13 [file 13065_2024_1179_MOESM1_ESM.doc]

**SUPPLEMENTRY MATERIAL FOR LIGAND 11 AND COBALT (II) COMPLEXES 12 AND 13**

[**Figure S1:** Powder X-ray diffractogram of compound **11** 2](#__RefHeading___Toc158291010)

[**Figure S2:** UV-VIS spectrum of compound **11** 2](#__RefHeading___Toc158291011)

[**Figure S3:** IR spectrum of compound **11** with some assignments 3](#__RefHeading___Toc158291012)

[**Figure S4:** HRESI+ mass spectrum of compound **11** 3](#__RefHeading___Toc158291013)

[**Figure S5:** ^1^H-NMR spectrum of compound **11** 3](#__RefHeading___Toc158291014)

[**Figure S6:** ^13^C-NMR spectrum of compound **11** 3](#__RefHeading___Toc158291015)

[**Figure S7:** HSQC spectrum of compound **11** 4](#__RefHeading___Toc158291016)

**[Figure S8:](../../../../I:/REVISION%2030%20JANVIER/3-BMCC-SUBMISSION-SM-Prof.SOPBUE_FEBUARY%202024_REVISED.doc#__RefHeading___Toc158291017)** COSY ^1^H-^1^H spectrum of compound **11** with some correlations 4

[**Figure S9:** HMBC spectrum of compound **11** 5](#__RefHeading___Toc158291018)

[**Figure S10:** UV-VIS spectrum of complex **12** 5](#__RefHeading___Toc158291019)

[**Figure S11:** IR spectrum of complex **12** 5](#__RefHeading___Toc158291020)

[**Figure S12:** HRESI+ mass spectrum of complex **12** 6](#__RefHeading___Toc158291021)

[**Figure S13:** ^1^H-NMR spectrum of complex **12** 6](#__RefHeading___Toc158291022)

[**Figure S14:** ^13^C-NMR spectrum of complex **12** 6](#__RefHeading___Toc158291023)

[**Figure S15:** HSQC spectrum of complex **12** with some correlations 7](#__RefHeading___Toc158291024)

[**Figure S16:** COSY spectrum of complex **12** 7](#__RefHeading___Toc158291025)

[**Figure S17:** HMBC spectrum of complex **12** with some correlations 7](#__RefHeading___Toc158291026)

[**Figure S18:** Comparison of the UV-VIS spectra of compound **11** (black) and **12** (blue) 8](#__RefHeading___Toc158291027)

[**Figure S19:** Comparison of the IR spectra of compounds **11** (black) and **12** (blue) 8](#__RefHeading___Toc158291028)

[**Figure S20**: Comparison of the ^1^H-NMR spectra of compounds **11** (**a**) and **12** (**b**) 9](#__RefHeading___Toc158291029)

[**Figure S21**: Comparison of the ^13^C-NMR spectra of compounds **11** (**a**) and **12** (**b**) 9](#__RefHeading___Toc158291030)

[**Figure S22:** Powder X-ray diffractogram of compound **13** 10](#__RefHeading___Toc158291031)

[**Figure S23:** UV-VIS spectrum of compound **13** 10](#__RefHeading___Toc158291032)

**[Figure S24:](../../../../I:/REVISION%2030%20JANVIER/3-BMCC-SUBMISSION-SM-Prof.SOPBUE_FEBUARY%202024_REVISED.doc#__RefHeading___Toc158291033)** IR spectrum of compound **13** 10

[**Figure S25:** HRESI+ mass spectrum of compound **13** 11](#__RefHeading___Toc158291034)

[**Figure S26:** ^1^H-NMR spectrum of compound **13** 11](#__RefHeading___Toc158291035)

[**Figure S27:** ^13^C-NMR spectrum of compound **13** 11](#__RefHeading___Toc158291036)

[**Figure S28:** HSQC spectrum of compound **13** with some correlations 12](#__RefHeading___Toc158291037)

[**Figure S29:** COSY spectrum of compound **13** 12](#__RefHeading___Toc158291038)

[**Figure S30:** HMBC spectrum of compound **13** with some correlations 12](#__RefHeading___Toc158291039)

[**Figure S31:** Comparison of the powder X-ray diffractograms of compounds **11** (black) and **13** (green) 13](#__RefHeading___Toc158291040)

[**Figure S32:** Comparison of the UV-VIS spectra of compound **11** (black) and **13** (green) 13](#__RefHeading___Toc158291041)

[**Figure S33:** Comparison of the IR spectra of compounds **11** (black) and **13** (green) 13](#__RefHeading___Toc158291042)

[**Figure S34**: Comparison of the ^1^H-NMR spectra of compound **11** (**a**) and **13 (b)** 14](#__RefHeading___Toc158291043)

[**Figure S35**: Comparison of the ^13^C-NMR spectra of compound **11** (**a**) and **13 (b)** 15](#__RefHeading___Toc158291044)

# **Figure S1:** Powder X-ray diffractogram of compound **11**

# **Figure S2:** UV-VIS spectrum of compound **11**


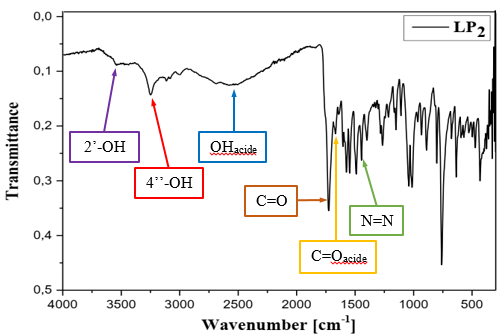


# **Figure S3:** IR spectrum of compound **11** with some assignments


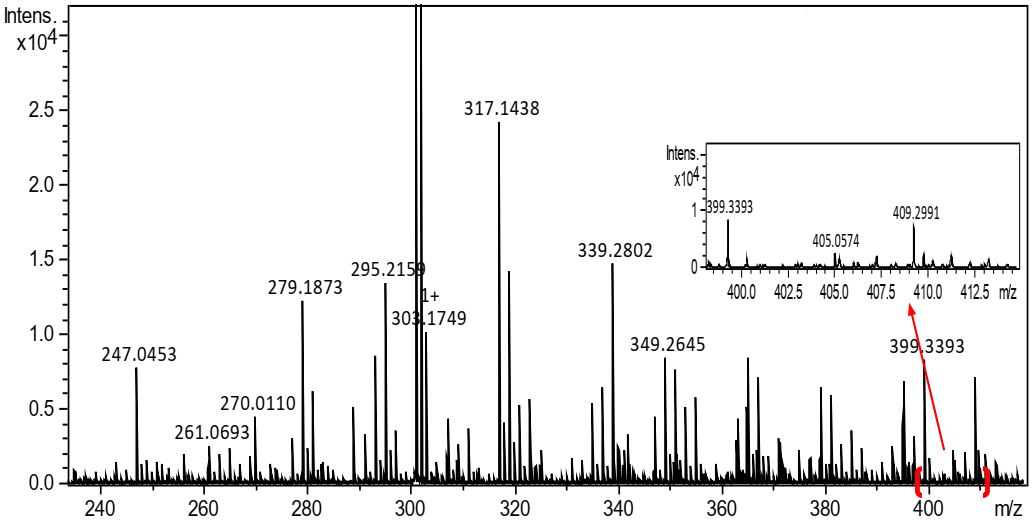


# **Figure S4:** HRESI+ mass spectrum of compound **11**


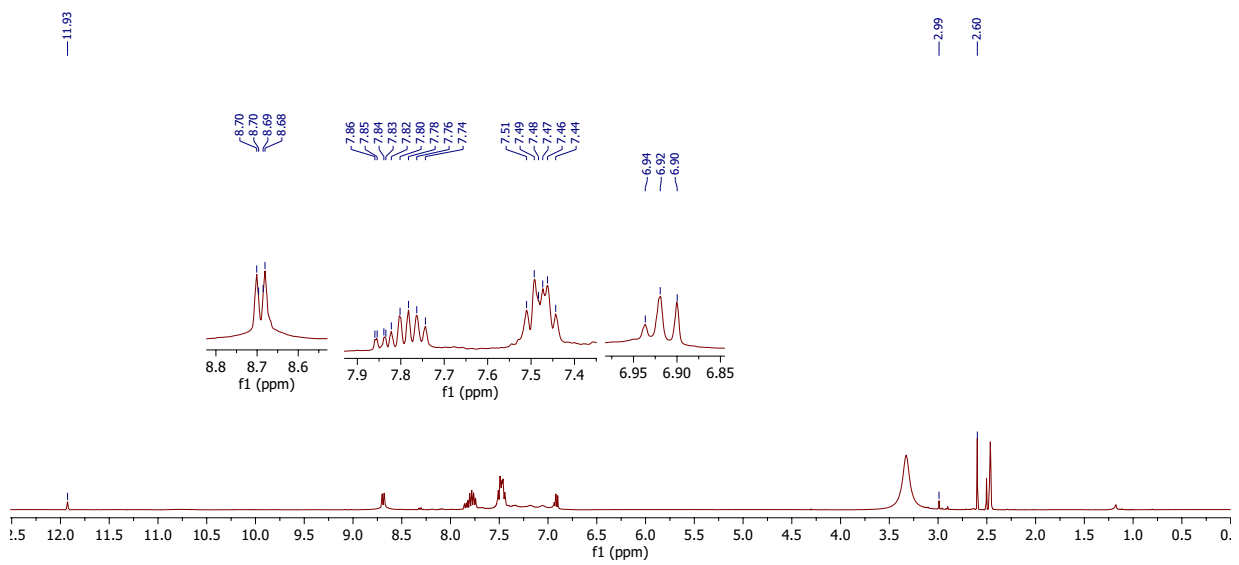


# **Figure S5:** ^1^H-NMR spectrum of compound **11**


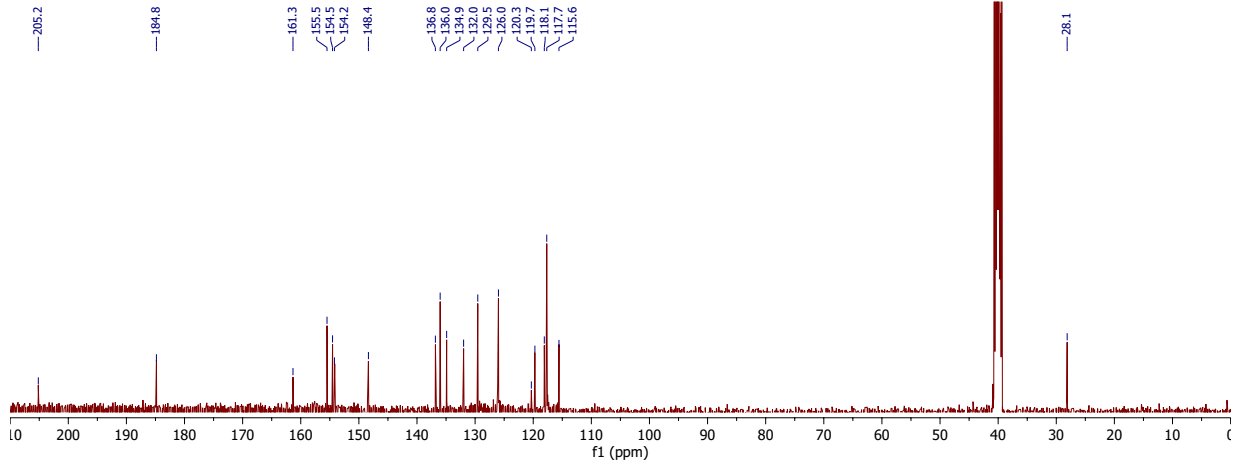


# **Figure S6:** ^13^C-NMR spectrum of compound **11**


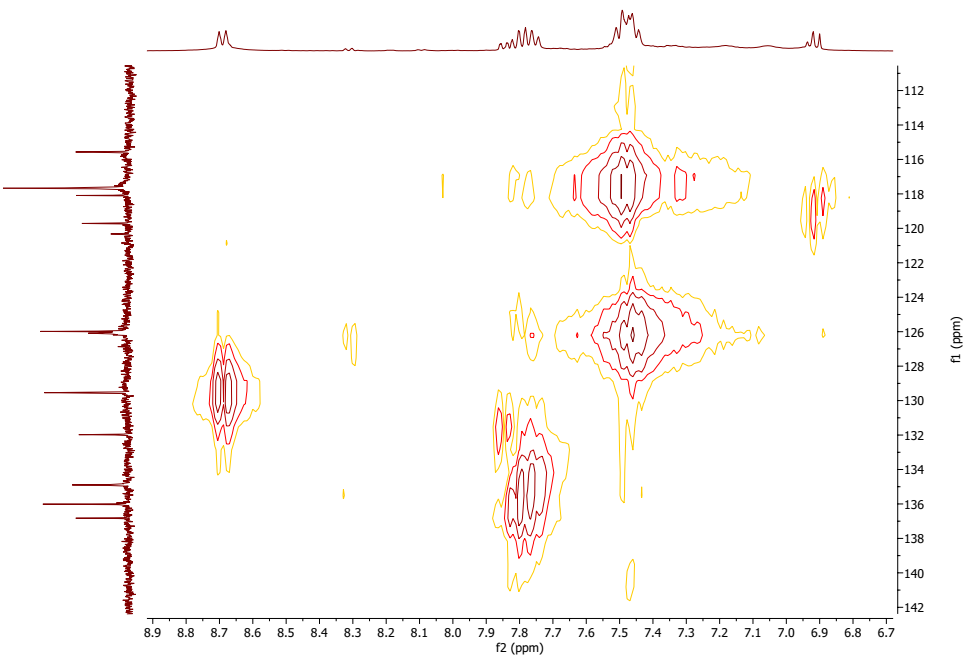


# **Figure S7:** HSQC spectrum of compound **11**


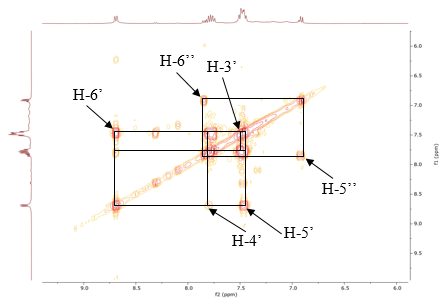


# **Figure S8:** COSY ^1^H-^1^H spectrum of compound **11** with some correlations


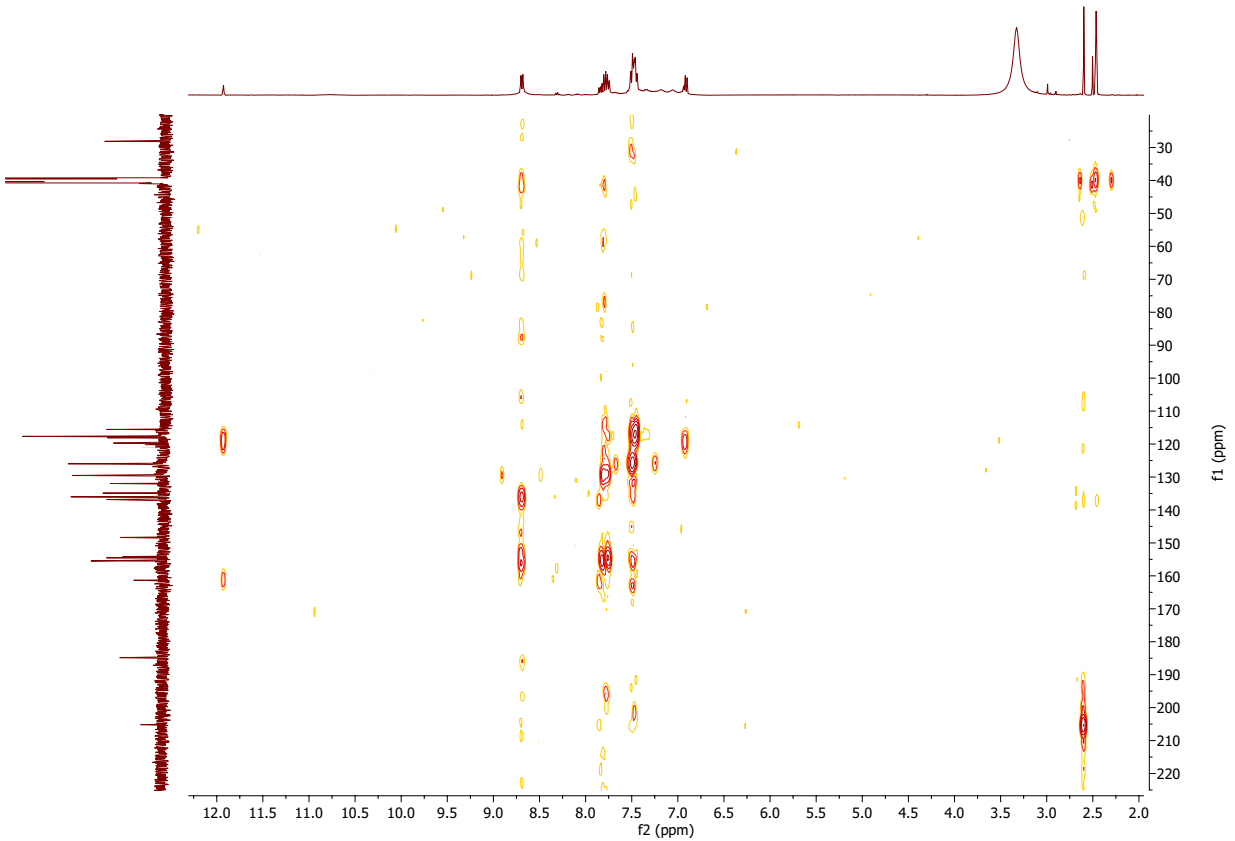


# **Figure S9:** HMBC spectrum of compound **11**


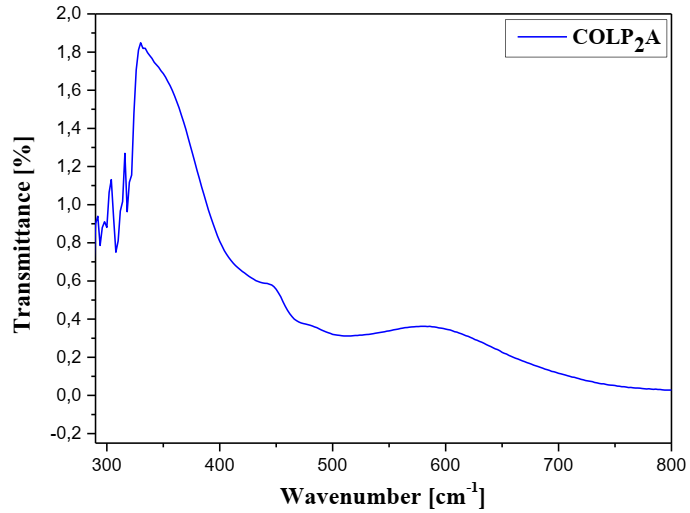


# **Figure S10:** UV-VIS spectrum of complex **12**


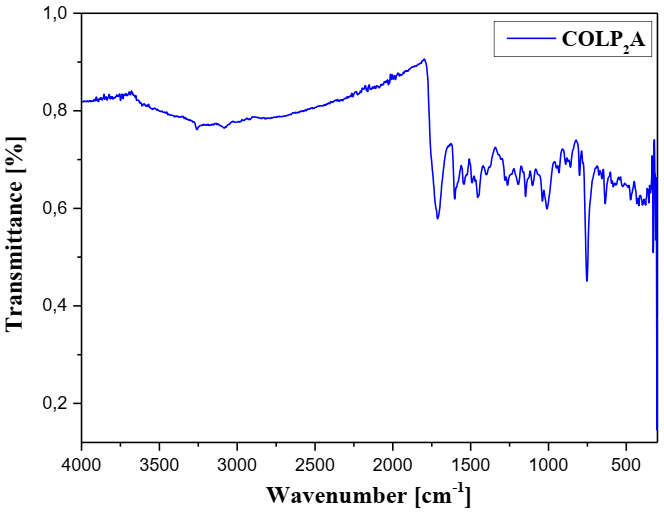


# **Figure S11:** IR spectrum of complex **12**


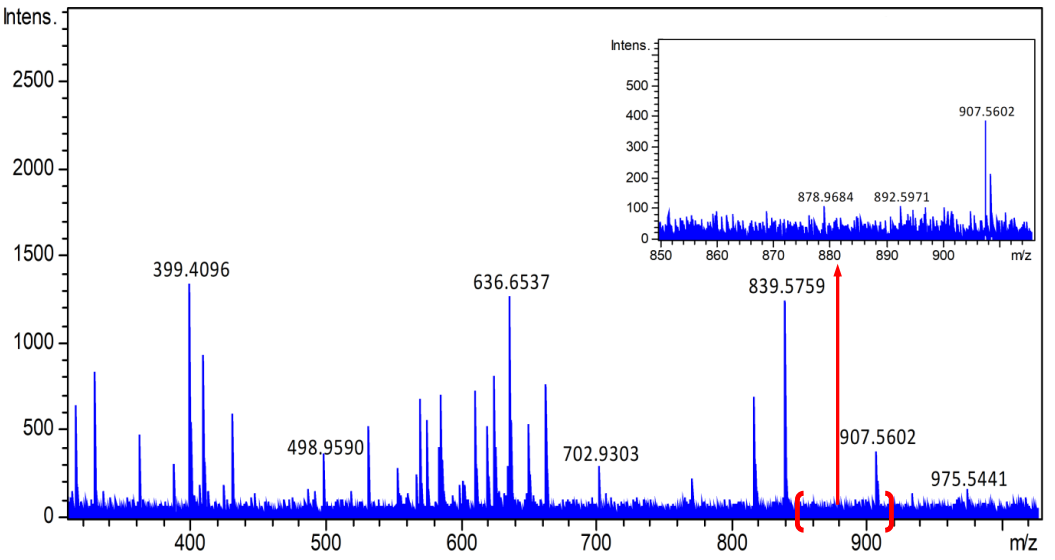


# **Figure S12:** HRESI+ mass spectrum of complex **12**


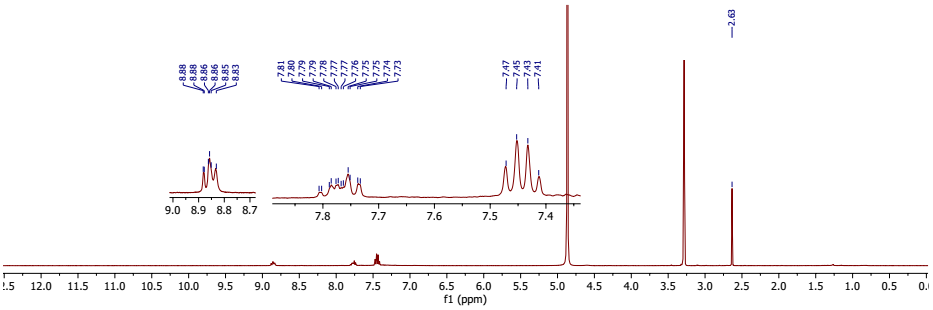


# **Figure S13:** ^1^H-NMR spectrum of complex **12**


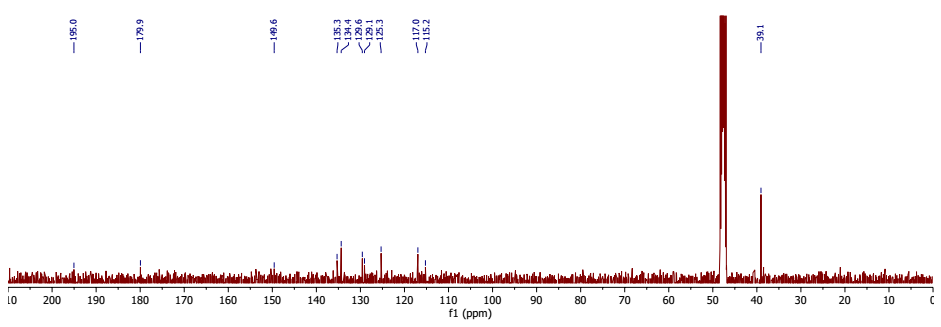


# **Figure S14:** ^13^C-NMR spectrum of complex **12**


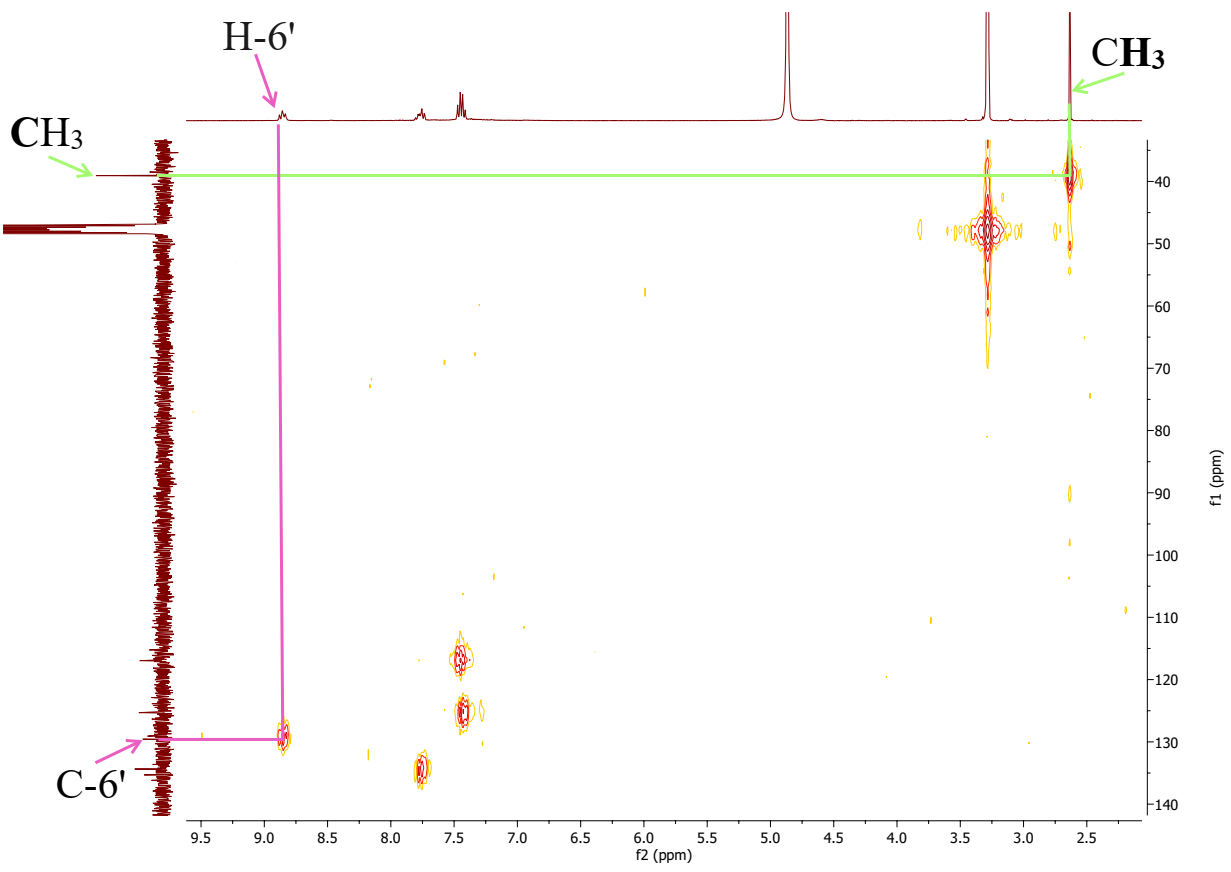


# **Figure S15:** HSQC spectrum of complex **12** with some correlations


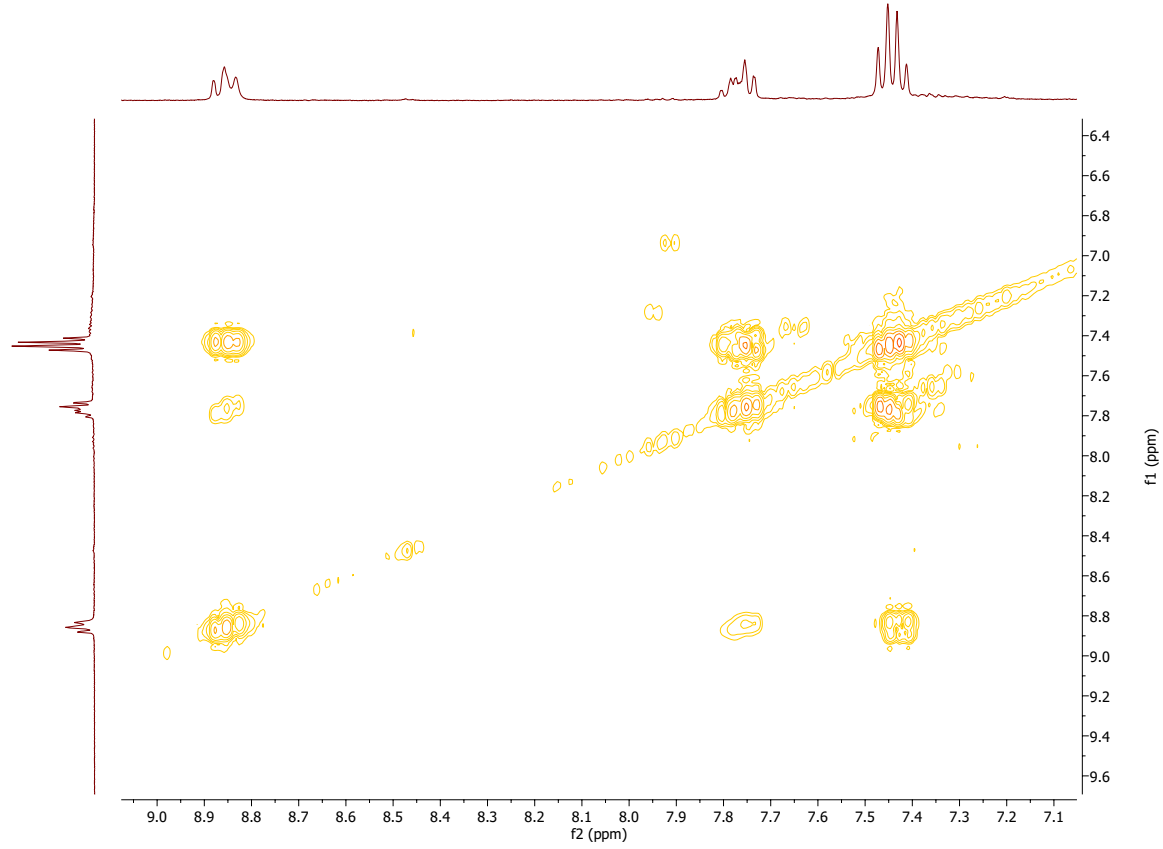


# **Figure S16:** COSY spectrum of complex **12**


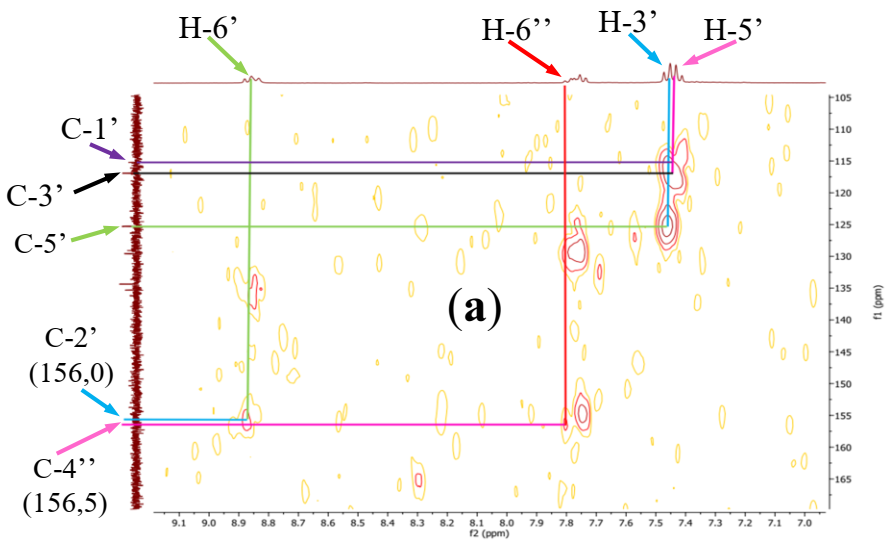


# **Figure S17:** HMBC spectrum of complex **12** with some correlations


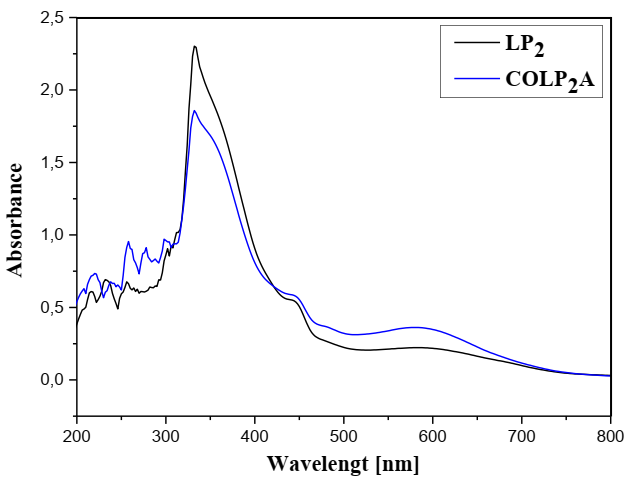


# **Figure S18:** Comparison of the UV-VIS spectra of compound **11** (black) and **12** (blue)


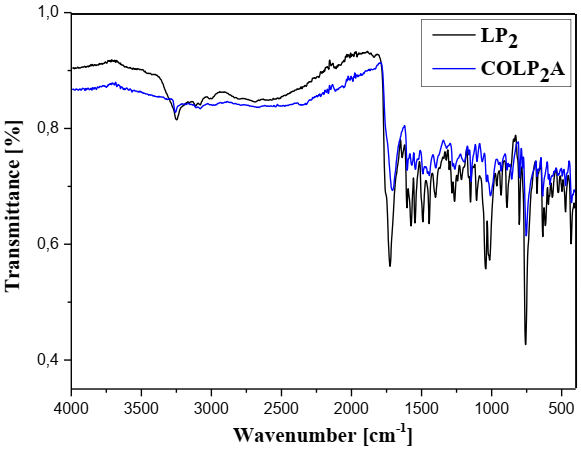


# **Figure S19:** Comparison of the IR spectra of compounds **11** (black) and **12** (blue)


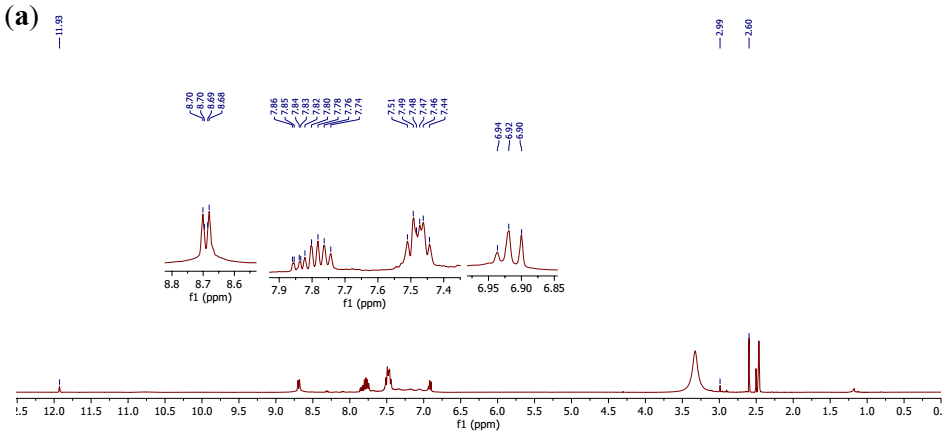


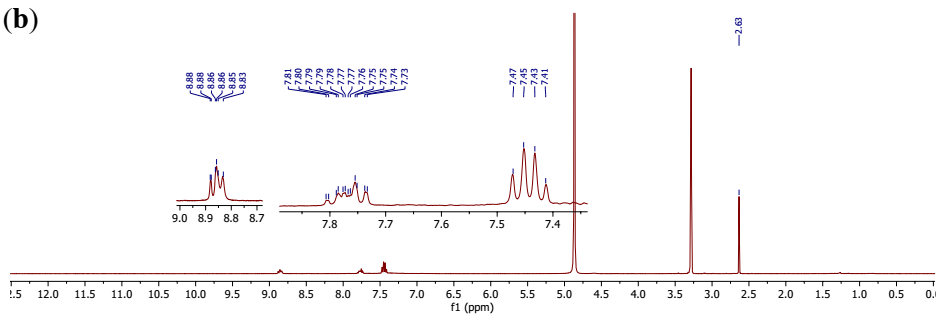


# **Figure S20**: Comparison of the ^1^H-NMR spectra of compounds **11** (**a**) and **12** (**b**)


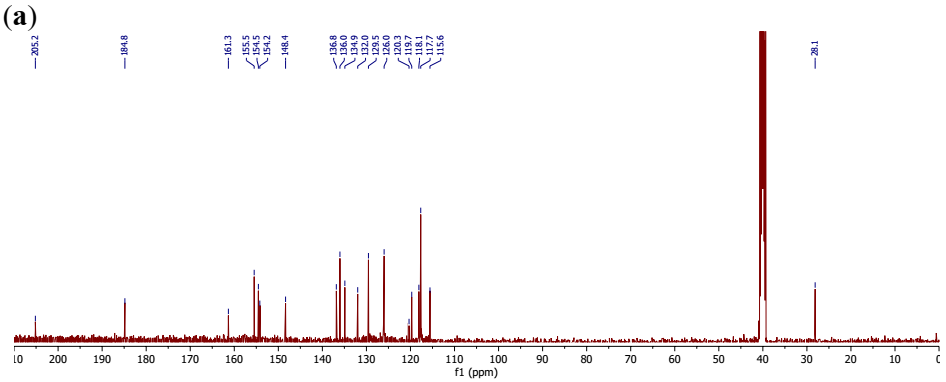


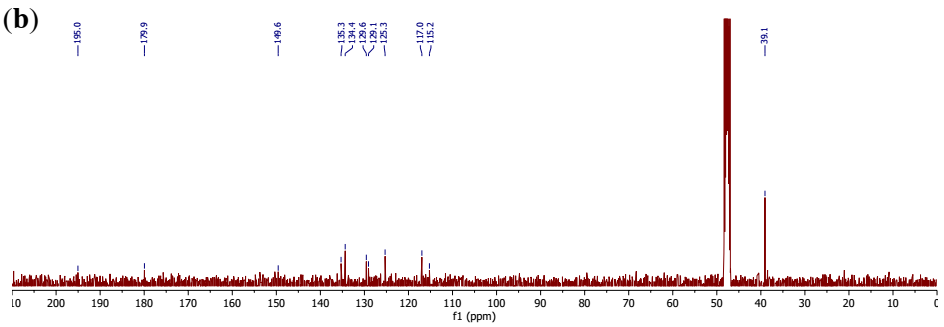


# **Figure S21**: Comparison of the ^13^C-NMR spectra of compounds **11** (**a**) and **12** (**b**)


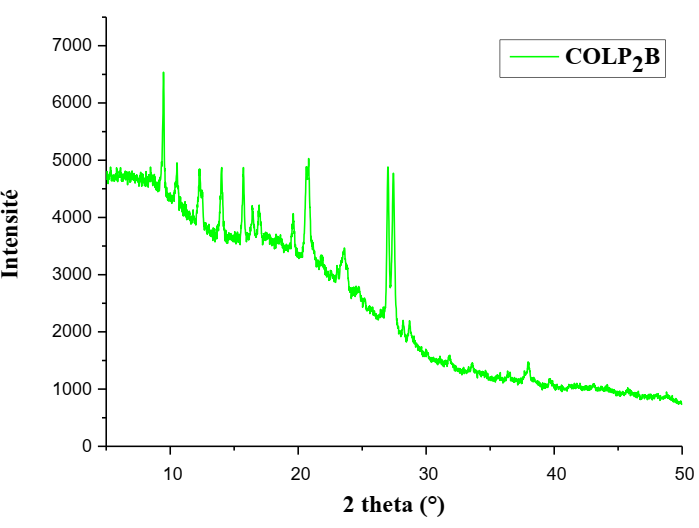


**6**

# **Figure S22:** Powder X-ray diffractogram of compound **13**


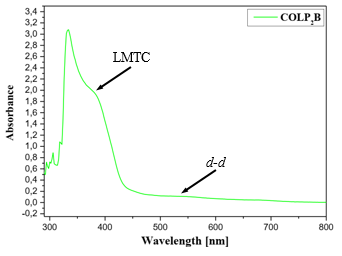


**6**

# **Figure S23:** UV-VIS spectrum of compound **13**


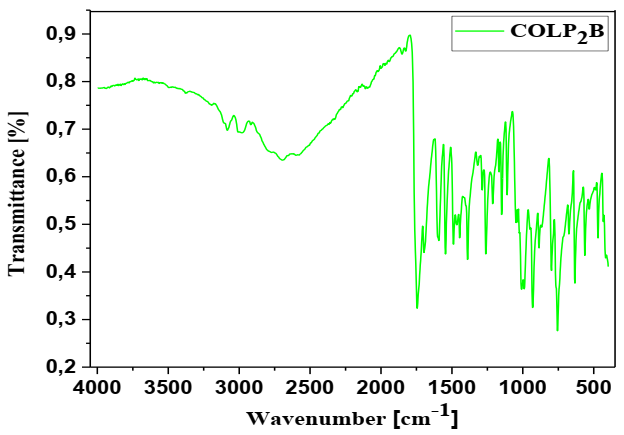


**6**

# **Figure S24:** IR spectrum of compound **13**


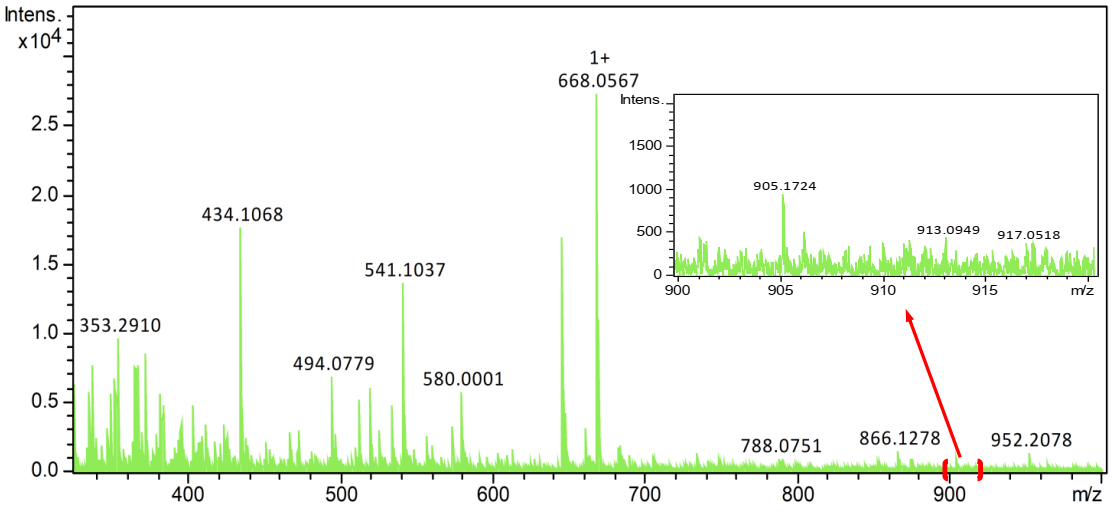


# **Figure S25:** HRESI+ mass spectrum of compound **13**


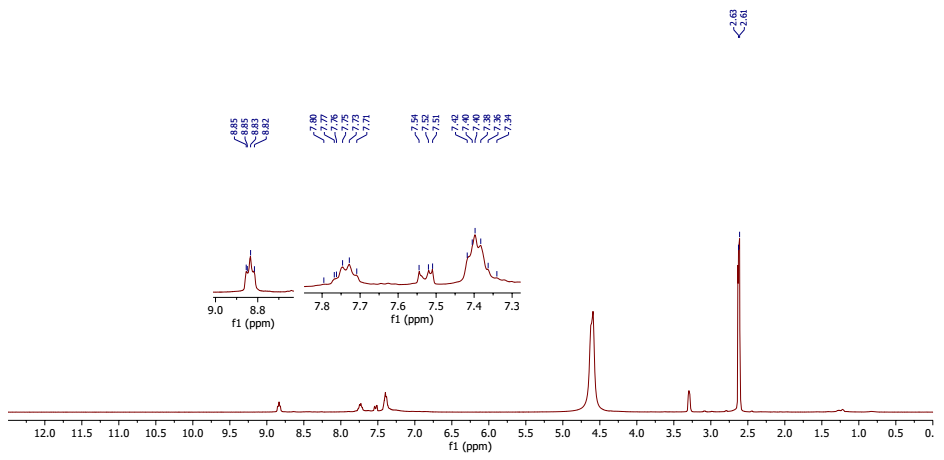


# **Figure S26:** ^1^H-NMR spectrum of compound **13**


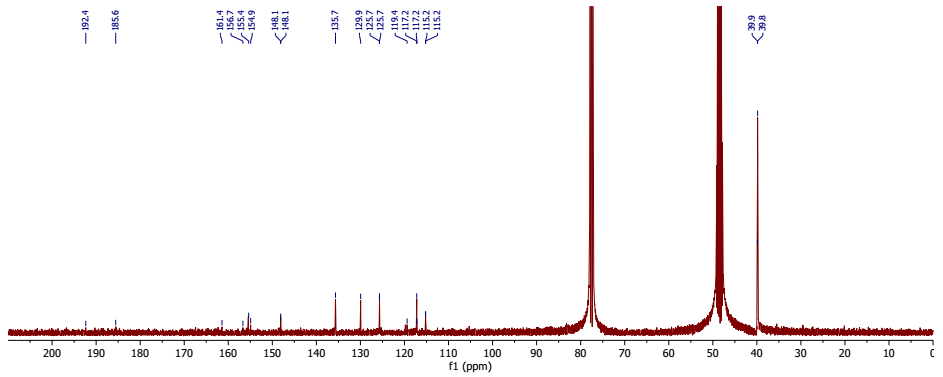


# **Figure S27:** ^13^C-NMR spectrum of compound **13**


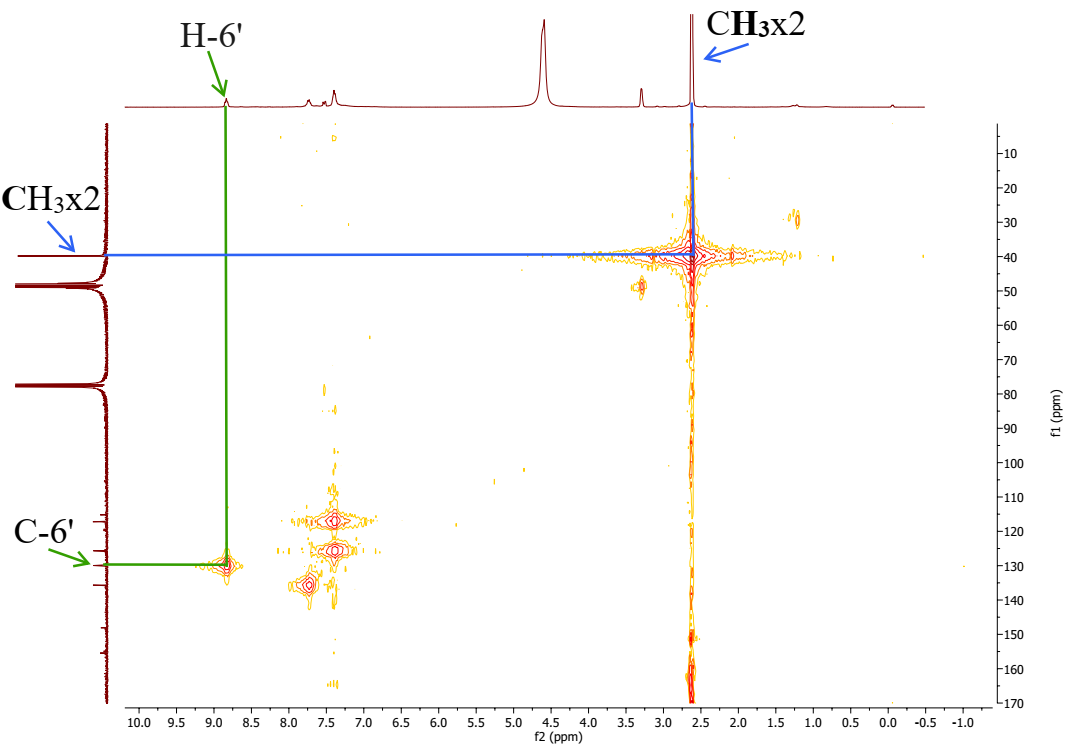


# **Figure S28:** HSQC spectrum of compound **13** with some correlations


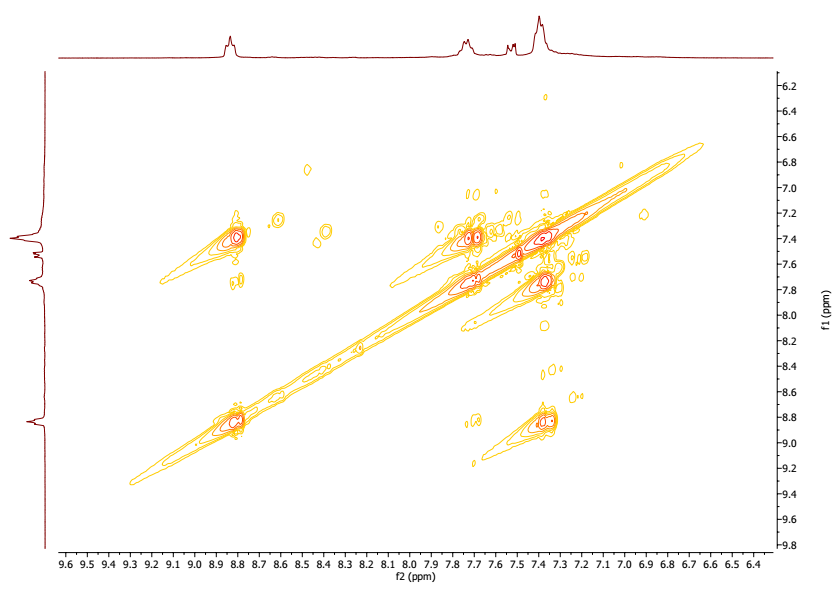


# **Figure S29:** COSY spectrum of compound **13**


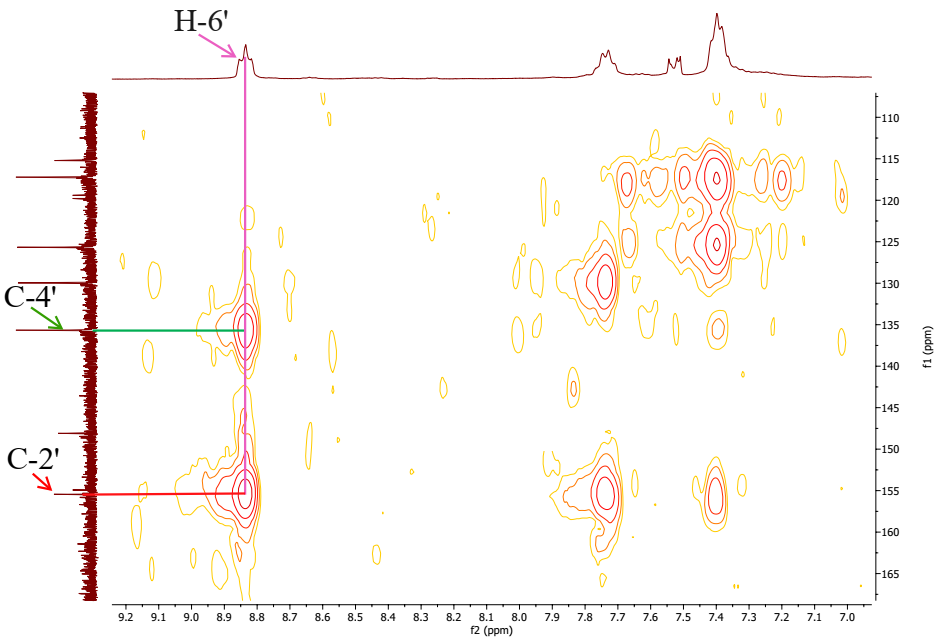


# **Figure S30:** HMBC spectrum of compound **13** with some correlations

# **Figure S31:** Comparison of the powder X-ray diffractograms of compounds **11** (black) and **13** (green)


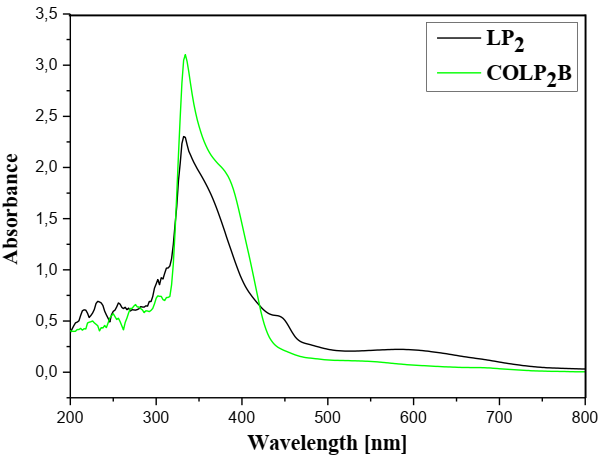


# **Figure S32:** Comparison of the UV-VIS spectra of compound **11** (black) and **13** (green)

# **Figure S33:** Comparison of the IR spectra of compounds **11** (black) and **13** (green)


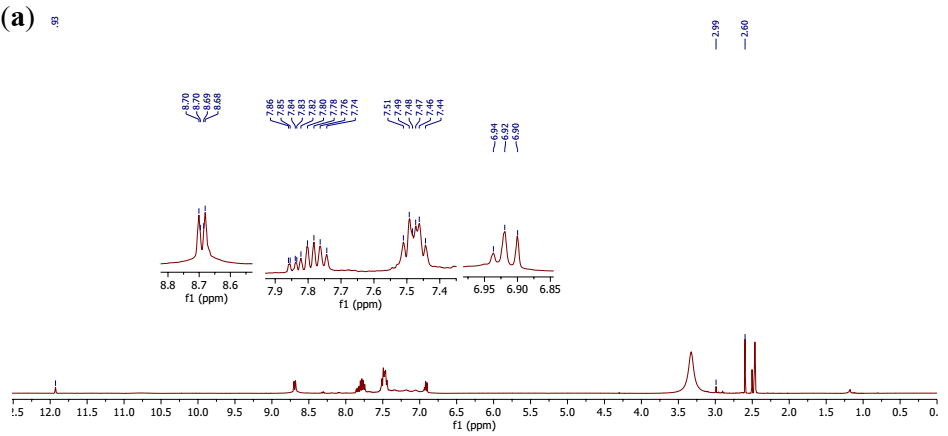


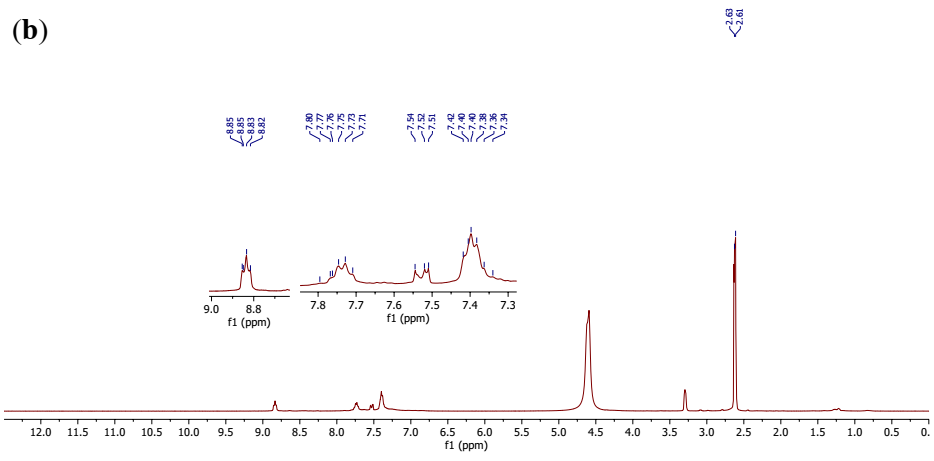


# **Figure S34**: Comparison of the ^1^H-NMR spectra of compound **11** (**a**) and **13 (b)**


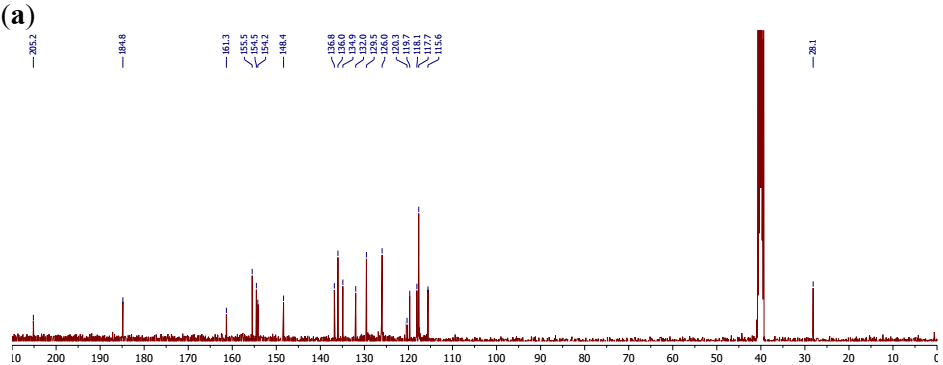


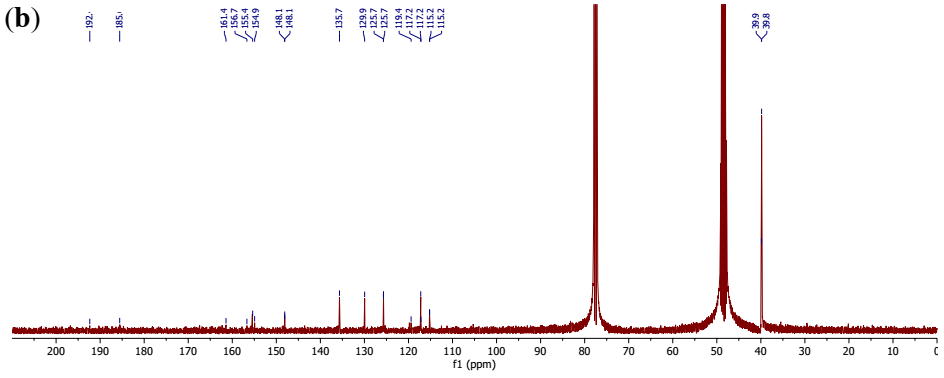


# **Figure S35**: Comparison of the ^13^C-NMR spectra of compound **11** (**a**) and **13 (b)**
